# Transformation Discriminant Analysis for Constructing Optimal Biomarker Combinations

**DOI:** 10.1002/sim.70665

**Published:** 2026-07-09

**Authors:** Ainesh Sewak, Sandra Siegfried, Torsten Hothorn

**Affiliations:** ^1^ Department of Clinical Research Universität Bern Bern Switzerland; ^2^ Institut für Epidemiologie, Biostatistik Und Prävention Universität Zürich Zürich Switzerland

**Keywords:** AUC, biomarker combination, biomarkers, classification, diagnostic tests, hepatocellular carcinoma, likelihood ratio, ROC

## Abstract

Accurate diagnostic tests are essential for effective screening and treatment. However, individual biomarkers often fail to provide sufficient diagnostic accuracy, as they typically capture only one aspect of the complex disease process. Combining multiple biomarkers, each capturing a distinct mechanism, can help construct more informative diagnostic tests. In practice, logistic regression is used as the default to combine biomarkers, but it can perform poorly when biomarker distributions exhibit skewness or differ across disease groups. Nonparametric methods provide more flexibility but generally require large sample sizes that are infrequently available in biomedical research. We propose a novel framework called transformation discriminant analysis which combines biomarkers through the likelihood ratio function to construct theoretically optimal diagnostic scores. Transformation discriminant analysis (TDA) balances between flexibility and efficiency. It can accommodate a wide range of distributional shapes and disease‐specific dependence structures while remaining fully parametric. This allows for likelihood inference and strong performance even in small‐sample settings. We evaluate TDA through simulations and benchmark its performance against commonly used methods. Finally, we illustrate its utility in constructing an optimal diagnostic test for hepatocellular carcinoma, a disease with no single ideal biomarker. An open‐source R implementation is provided for reproducibility and broader application.

## 1 Introduction

Diagnostic testing is central to modern healthcare because it enables the timely identification and management of diseases. Most diagnostic tests rely on individual biomarkers for screening and subsequent treatment decisions [1]. However, the biological heterogeneity of many diseases means that single biomarkers often fail to capture the full picture. This has driven a shift in precision medicine towards using biomarker panels, where multiple markers used together can offer a more comprehensive view of disease pathology [2]. Statistically combining these markers effectively is key to improving diagnostic accuracy.

What is the best way to combine information from multiple biomarkers to discriminate diseased from nondiseased populations? Methodological research has largely focused on linear combinations. Early approaches assumed multivariate normality, leading to solutions like discriminant analysis [3]. Later methods relaxed this assumption and optimized empirically based on performance metrics [4, 5, 6, 7]. However, linear combinations are not necessarily optimal. They may struggle to capture interactions between biomarkers or distributional differences between disease populations [8].

Theoretically, the path to optimality is clear: the likelihood ratio function provides the uniformly most powerful decision rule for binary classification [9, 10]. Further, any monotonic transformation, such as a risk score, retains this optimality [11]. This has led to logistic regression becoming a default method in practice. However, when data are skewed or exhibit disease‐specific dependence structures, logistic regression can yield biased estimates and suboptimal performance [12]. While the likelihood ratio remains a viable solution, a gap remains between this theoretical ideal and its practical implementation. Estimating multivariate distributions required for the likelihood ratio is mathematically and computationally challenging [13].

We address this gap by proposing a framework we term transformation discriminant analysis (TDA), adopting terminology introduced previously [14]. We develop a flexible multivariate transformation model that estimates the joint distributions of biomarkers and through which we can construct an optimal composite score via the likelihood ratio function. TDA generalizes LDA by operating on a transformed scale and accommodates key clinical complexities such as skewed marginals, disease‐specific dependence structures and missing biomarkers. Its parameterization supports efficient computation of diagnostic metrics such as ROC curves and AUC, along with standard likelihood‐based inference. We further develop model assessment techniques to evaluate goodness‐of‐fit of the underlying model.

We demonstrate TDA's utility in the context of hepatocellular carcinoma (HCC), the most common form of liver cancer. Current diagnostic practices for HCC rely on imaging, biopsy, and serum biomarkers. Among these, alpha‐fetoprotein (AFP) is most commonly used, but its standalone diagnostic performance is limited, especially in patients with benign liver conditions [15]. To address this limitation, alternative biomarkers have been proposed [16]. Using data from a retrospective case–control study [17], we construct and evaluate an optimal diagnostic score that combines AFP with additional biomarkers to improve HCC detection.

In the sections that follow, we first introduce the notation and briefly review existing methods for combining multiple biomarkers. We then present the TDA framework, describing the multivariate transformation model, the associated likelihood ratio function, special cases, connections to existing models and estimation procedures. The method's performance is evaluated through simulation studies, followed by an application to hepatocellular carcinoma diagnosis. We discuss the results and outline future directions. We conclude by introducing an R add‐on package that implements TDA and provides reproducibility materials.

## 2 Optimal Biomarker Combinations

### 2.1 Notation

We focus on data derived from case–control study designs, where D represents a binary random variable indicating the absence (D=0, denoting a nondiseased subject) or presence (D=1) of a specific disease, such as histologically confirmed HCC from our application. The random vector $Y_{d}=\left\{Y|D=d\right\}={\left(Y_{d1},Y_{d2},\ldots ,Y_{\mathrm{dJ}}\right)}^{⊤}\in {\mathbb{R}}^{J}$ represents the J absolutely continuous biomarker observations of a subject with disease status D=d. Let $f_{d}:{\mathbb{R}}^{J}\mapsto {\mathbb{R}}^{+}$ be the absolutely continuous joint conditional probability density function (PDF) of the biomarkers, with $f_{0}$ characterizing the biomarker PDF for the nondiseased population and $f_{1}$ for the diseased population. The development of an optimal diagnostic score is based on data obtained from independent observations, denoted as $i=1,\ldots ,N=N_{0}+N_{1}$, originating from both nondiseased and diseased populations.

The primary objective of this paper is to derive a scalar‐valued function, denoted by $L:{\mathbb{R}}^{J}\mapsto \mathbb{R}$. This function combines the J biomarkers into a composite diagnostic score and we aim at finding a function L maximizing diagnostic accuracy. The composite score can be employed to classify a yet undiagnosed subject based on observed biomarker values $y=\left(y_{1},\ldots ,y_{J}\right)\in {\mathbb{R}}^{J}$. The classification involves designating a subject as diseased when L(y)>c for a specified threshold c∈ℝ.

Define $L_{0}=\log\left(L\left(Y_{0}\right)\right)∼G_{0}$ and $L_{1}=\log\left(L\left(Y_{1}\right)\right)∼G_{1}$ as the log‐transformed random variables of the resulting composite scores in the nondiseased and diseased populations, with $G_{0}$ and $G_{1}$ being their respective absolute continuous cumulative distribution functions (CDFs). Specificity and sensitivity, representing the probability of accurately identifying nondiseased or diseased subjects, are defined by $\mathbb{P}\left(L_{0}\leq c\right)=G_{0}\left(c\right)$ and $\mathbb{P}\left(L_{1}>c\right)=1-G_{1}\left(c\right)$. The ROC curve graphically summarizes the trade‐off between sensitivity and specificity, serving as a quantifiable measure for diagnostic accuracy in an optimal test. The optimal ROC curve, based on the composite score, is given by $\mathrm{ROC}\left(p\right)=1-G_{1}\left(G_{0}^{-1}\left(1-p\right)\right)$.

### 2.2 Related Work

Various approaches have been proposed for developing combinations of multiple biomarkers for discriminating between two populations. Linear combinations of biomarkers aim to find the best set of coefficients $a\in {\mathbb{R}}^{J}$ such that the composite diagnostic scores $L\left(Y_{d}\right)=a^{⊤}Y_{d}$ maximize discrimination between disease populations. Under the assumption of multivariate normality for $Y_{d}$, the classical linear discriminant analysis (LDA) coefficient yields the optimal linear combination [3]. To avoid distributional assumptions, later methods focused on optimizing empirical performance criteria such as the AUC [4, 13, 18], partial AUC [12, 19], or the Youden index [6]. These methods are flexible and provide distribution‐free linear combinations of biomarkers. However, linearity imposes some limitations. Such scores may fail to capture skewed biomarker distributions, interactions or nonlinearities that often arise in biomedical applications [8].

From a theoretical perspective, the likelihood ratio function

$$L\left(Y_{d}\right)=\frac{f_{1}\left(Y_{d}\right)}{f_{0}\left(Y_{d}\right)},$$

provides the optimal combination under the Neyman‐Pearson lemma [11]. When used as a composite diagnostic score, the resulting ROC curve maximizes sensitivity at every level of specificity [20]. All associated performance criteria such as the AUC, pAUC and Youden Index are also maximized. By Bayes' theorem, the likelihood ratio is also a monotone function of the posterior odds of disease

(1) $$\log\left(\frac{\mathbb{P}\left(D=1|Y=y\right)}{\mathbb{P}\left(D=0|Y=y\right)}\right)=\log\left(\frac{\mathbb{P}\left(D=1\right)}{\mathbb{P}\left(D=0\right)}\right)+\log\left(\frac{f_{1}\left(y\right)}{f_{0}\left(y\right)}\right).$$

This decomposition motivates two modeling strategies. *Discriminative* methods, such as logistic regression, model the left‐hand side directly, typically by imposing a linear structure on the log‐odds of disease. More flexible machine learning methods can also be used to estimate ℙ(D=1|Y) directly [18], though they often require large sample sizes and lack interpretability.

*Generative* methods instead model the disease‐specific densities $f_{0}$ and $f_{1}$. LDA is a generative method and when correctly specified, it can be more statistically efficient. For example, logistic regression is only two‐thirds as efficient as LDA under multivariate normality [21] and some have advocated against the default use of logistic regression due to its inefficiency in nonnormal settings [22]. Nevertheless, logistic regression remains widely used due to its simplicity, robustness across many settings (e.g., exponential families) and interpretability [23]. However, it cannot accommodate nonnormal settings or disease‐specific dependence structures unless explicitly extended [24].

Beyond the multivariate normal setting, flexible estimation of the full joint densities $f_{0}$ and $f_{1}$ is challenging. Some authors therefore model the likelihood ratio directly to circumvent this difficulty [25, 26, 27]. Others use generative semiparametric approaches that first map biomarkers to an approximately normal scale via ${\hat{h}}_{j}\left(x\right)=\Phi^{-1}\left({\hat{F}}_{j}\left(x\right)\right)$, and then estimate the dependence structure on the transformed scale. The earliest such approach, also termed TDA, transforms each variable using empirical rank‐based quantiles, estimates class means and variances through approximate method‐of‐moments estimators and in a separate step the dependence structure via Van der Waerden normal score rank correlations [14]. Related semiparametric normal‐score and nonparanormal classifiers were later developed in the machine‐learning literature [28]. More recent methods use semiparametric transformation models estimated sequentially rather than through joint likelihood maximization [29, 30]. The most recent contribution estimates the full joint density nonparametrically using smoothing splines [31], but requires bandwidth selection and does not directly support inference for diagnostic accuracy measures such as the AUC. Across these approaches, uncertainty in marginal estimation is typically not propagated into the dependence structure, and standard uncertainty quantification for model parameters or diagnostic performance criteria is generally unavailable.

These limitations motivate the framework developed in the remainder of this paper. TDA models disease‐specific joint biomarker distributions through a multivariate transformation model which can flexibly capture marginal distributions and disease‐specific dependence structures. The resulting likelihood ratio defines composite scores that are optimal under the Neyman‐Pearson paradigm when correctly specified. TDA is fully parametric and jointly maximizes a single likelihood over all model parameters, including the marginal transformations and dependence structure simultaneously. This enables likelihood‐based inference for both model parameters and diagnostic accuracy measures such as the AUC and ROC curve. This parametric structure also naturally accommodates incomplete biomarker measurements through the observed‐data likelihood. Particularly in small‐sample biomedical applications, where nonparametric approaches tend to exhibit high variance and unreliable tail behavior, our method balances flexibility with parametric efficiency.

## 3 Multivariate Transformation Model

We propose a multivariate transformation model featuring an unknown transformation function $h_{d}:{\mathbb{R}}^{J}\mapsto {\mathbb{R}}^{J}$ to model the joint density and account for the correlation between biomarkers [32]. This function is defined coordinate‐wise on the observed biomarkers, $h_{d}\left(y\right)={\left(h_{d1}\left(y_{1}\right),\ldots ,h_{\mathrm{dJ}}\left(y_{J}\right)\right)}^{⊤}$ and is monotonically nondecreasing in each of its coordinates.

The purpose of this transformation is to map the unknown distribution of $Y_{d}$ for a given disease indicator D=d∈{0,1} to a random vector with a known distribution, denoted as $Z_{d}=h_{d}\left(Y_{d}\right)$. Specifically, the vector $Z_{d}={\left(Z_{d1},\ldots ,Z_{\mathrm{dJ}}\right)}^{⊤}$ follows a zero‐mean multivariate normal distribution, $Z_{d}∼N_{J}\left(0,\Sigma_{d}\right)$, with a disease‐dependent covariance matrix $\Sigma_{d}\in {\mathbb{R}}^{J\times J}$. The entries of the covariance matrix measure the dependence between the *transformed* biomarkers in each of the populations. The joint CDF of $Y_{d}$ is given by

$$\begin{array}{l} \mathbb{P}\left(Y\leq y|D=d\right)=\mathbb{P}\left(Y_{d}\leq y\right)=\mathbb{P}\left(h_{d}\left(Y_{d}\right)\leq h_{d}\left(y\right)\right)\\ =\mathbb{P}\left(Z_{d}\leq h_{d}\left(y\right)\right)=\Phi_{0,\Sigma_{d}}\left(h_{d1}\left(y_{1}\right),\ldots ,h_{\mathrm{dJ}}\left(y_{J}\right)\right) \end{array}$$

where $\Phi_{0,\Sigma }$ is the joint CDF of a multivariate normal distribution with a zero mean vector and covariance matrix Σ. To ensure identifiability, we standardize this matrix such that $\mathrm{diag}\left(\Sigma_{d}\right)=1$ and $\Sigma_{d}$ is a correlation matrix. This leads to the interpretation of $h_{\mathrm{dj}}$ as marginal distribution functions on the probit scale $\mathbb{P}\left(Y_{j}\leq y_{j}|D=d\right)=\mathbb{P}\left(Y_{\mathrm{dj}}\leq y_{j}\right)=\Phi \left(h_{\mathrm{dj}}\left(y_{j}\right)\right)$. The following proposition provides the optimal function for combining multiple biomarkers under the multivariate transformation model.

Proposition 1 *Suppose the derivatives of the marginal transformation functions exist such that* $h_{\mathrm{dj}}^{\prime }\left(y_{j}\right)>0$ *for* j=1,…,J *and let the joint PDF of the biomarkers be*

$$f_{d}\left(y\right)=\phi_{0,\Sigma_{d}}\left(h_{d1}\left(y_{1}\right),\ldots ,h_{\mathrm{dJ}}\left(y_{J}\right)\right)\prod_{j=1}^{J}h_{\mathrm{dj}}^{\prime }\left(y_{j}\right),$$

*where* $\phi_{0,\Sigma }$ *is the joint PDF of a multivariate normal distribution with a zero mean vector and correlation matrix* Σ. *Then the log‐likelihood ratio function is*

$$\begin{array}{cc} \log\left(L\left(y\right)\right) & =-\frac{1}{2}\left(\log\left(\frac{\left|\Sigma_{1}\right|}{\left|\Sigma_{0}\right|}\right)+h_{1}{\left(y\right)}^{⊤}\Sigma_{1}^{-1}h_{1}\left(y\right)-h_{0}{\left(y\right)}^{⊤}\Sigma_{0}^{-1}h_{0}\left(y\right)\right)\\ & \;+\sum_{j=1}^{J}\log\left(\frac{h_{1j}^{\prime }\left(y_{j}\right)}{h_{0j}^{\prime }\left(y_{j}\right)}\right), \end{array}$$

*where* $|\Sigma_{d}|\neq 0$ *is the determinant of the matrix* $\Sigma_{d}$.

The proof of Proposition 1 is given in Appendix B and follows from the definition of the likelihood ratio function. We call all ROC curves and AUCs derived from the likelihood ratio function under the multivariate transformation model as *model‐based* ROC curves and AUCs.

### 3.1 Location‐Scale Marginal Model

The general multivariate transformation model, as presented above, incorporates fully flexible marginal distributions for the biomarkers in each of the nondiseased and diseased classes. This model requires simulations for the sampling distributions of $L_{0}$ and $L_{1}$, particularly when calculating the optimal model‐based ROC curve and corresponding AUC, given the absence of simple closed‐form expressions. We give a location‐scale simplification of the marginal model in the following proposition which ensures analytical accessibility to these distributions, while requiring fewer overall model parameters.

Proposition 2 *Assume a common transformation function* $h:{\mathbb{R}}^{J}\mapsto {\mathbb{R}}^{J}$ *with* $h\left(y\right)={\left(h_{1}\left(y_{1}\right),\ldots ,h_{J}\left(y_{J}\right)\right)}^{⊤}$ *such that the* j *th marginal transformation function is defined as*.

$$h_{\mathrm{dj}}\left(y_{j}\right)=\frac{h_{j}\left(y_{j}\right)-\delta_{j}d}{\exp\left(\gamma_{j}d\right)}\;\text{for}\;j=1,\ldots ,J,$$

*where* $\delta_{j}\in \mathbb{R}$ *and* $\exp\left(\gamma_{j}\right)\in {\mathbb{R}}^{+}$. *Then the multivariate model can be expressed as*

$$h\left(Y_{d}\right)=\delta_{d}+\Gamma_{d}^{-1}Z_{d},$$

*where* $\delta_{0}=0$, $\delta_{1}=\delta ={\left(\delta_{1},\ldots ,\delta_{J}\right)}^{⊤}$, $\Gamma_{0}^{-1}=I$, $\Gamma_{1}^{-1}=\Gamma^{-1}=\mathrm{diag}\left(\exp\left(\gamma_{1}\right),\ldots ,\exp\left(\gamma_{J}\right)\right)$, $Z_{d}∼N_{J}\left(0,\Sigma_{d}\right)$ *and the log‐likelihood ratio function is*

$$\log\left(L\left(y\right)\right)\propto {\left(h\left(y\right)-\beta \right)}^{⊤}A\left(h\left(y\right)-\beta \right),$$

*where* $A={\Gamma \Sigma }_{1}^{-1}\Gamma -\Sigma_{0}^{-1}$ *and* $\beta =\left(I+{\left(\Sigma_{0}A\right)}^{-1}\right)\delta$.

The proof of Proposition 2 is given in Appendix B and follows from Proposition 1. The parameters within the marginal models are also interpretable as follows. The location term $\delta_{j}$ accommodates distinct baseline biomarker levels for diseased and nondiseased cases while $\exp\left(\gamma_{j}\right)$ represents the scaling term allowing for different degrees of dispersion based on disease status. Including such a scale term substantially widens the class of marginal distributions that can be represented [33].

The following corollary directly arises from Proposition 2 and allows for fast computation of diagnostic accuracy metrics for the composite score $L_{d}$, including model‐based ROC curves and AUCs. This approach involves evaluating a univariate generalized chi‐square distribution, defined as a weighted sum of non‐central chi‐square distributions [34], whose parameters are derived from the coefficients of the location‐scale multivariate model.

Corollary 1 *Let the spectral decomposition of* ${\tilde{\Sigma }}_{d}^{\frac{1}{2}}A{\tilde{\Sigma }}_{d}^{\frac{1}{2}}$ *be given by* $P_{d}W_{d}P_{d}^{⊤}$ *where* ${\tilde{\Sigma }}_{d}$ *is the correlation matrix of* $h\left(Y_{d}\right)$. *Then the scalar composite score* $L_{d}$ *follows a generalized chi‐square distribution* $G_{\chi_{J}^{2}}\left(w_{d},\nu_{d}\right)$ *with weights as* $w_{d}=\mathrm{diag}\left(W_{d}\right)$, *the non‐centrality parameters* $\nu_{d}=\mathrm{diag}{\left(P_{d}^{⊤}\Sigma_{d}^{-\frac{1}{2}}\left(\delta_{d}-\beta \right)\right)}^{2}$ *and the degrees of freedom* $1\in {\mathbb{R}}^{J}$.

Note that $P_{d}$ is an orthogonal matrix whose columns are the real, orthonormal eigenvectors and $W_{d}$ is a diagonal matrix whose entries are the eigenvalues. The proof for this result can be found in Appendix B. It follows from the fact that the log‐likelihood ratio function under this model takes a quadratic form and thus the resulting distribution of the scalar composite score $L_{d}$ is also in a quadratic form of a multivariate normal variable.

The generalized chi‐square distribution lacks a closed‐form distribution function, but efficient computational methods have been developed for its evaluation [35]. Therefore, as the optimal model‐based ROC curve is defined by the distribution functions of $L_{0}$ and $L_{1}$, it can be calculated directly from the model parameters, along with the corresponding AUC.

### 3.2 Relationship to LDA

In a specific case of our model, we arrive at the same result as LDA, which is established as the optimal linear combination of biomarkers [3]. However, our approach extends this result to include combinations of transformed biomarkers without imposing the assumption of normality on $Y_{d}$.

Corollary 2 *Assume a global covariance matrix* $\Sigma_{d}=\Sigma$ *for both classes, with a single multivariate transformation function* $h_{d}=h$, *and omitting scaling terms* $\Gamma_{d}=I$ *for* d=0,1. *Then the log‐likelihood ratio function is* $\delta^{⊤}\Sigma^{-1}\left(h\left(y\right)-\frac{1}{2}\delta \right)$.

Given that the log‐likelihood ratio is linear in the vector of transformed biomarkers h(y), the best linear combination of the transformed biomarkers is proportional to the Fisher's discriminant coefficient $\delta^{⊤}\Sigma^{-1}$. In this model, the distribution of the transformed biomarkers in the nondiseased class is given by $h\left(Y_{0}\right)∼N_{J}\left(0,\Sigma \right)$, and in the diseased class, it is $h\left(Y_{1}\right)∼N_{J}\left(\delta ,\Sigma \right)$. Consequently, the distributions of the composite scores are $L_{0}∼N\left(-\frac{1}{2}\delta^{⊤}\Sigma^{-1}\delta ,\delta^{⊤}\Sigma^{-1}\delta \right)$ and $L_{1}∼N\left(\frac{1}{2}\delta^{⊤}\Sigma^{-1}\delta ,\delta^{⊤}\Sigma^{-1}\delta \right)$. The optimal model‐based ROC curve is binormal and can be expressed as

(2) $$\mathrm{ROC}\left(p\right)=1-\Phi \left(\Phi^{-1}\left(1-p\right)-\sqrt{\delta^{⊤}\Sigma^{-1}\delta }\right),$$

while the AUC is given by

(3) $$\mathrm{AUC}=\Phi \left(\sqrt{\frac{\delta^{⊤}\Sigma^{-1}\delta }{2}}\right).$$

In practice, and also in our application, the differences between the biomarker distributions of diseased and nondiseased cases often extend beyond linear location shifts, even on the transformed scale defined by h. This necessitates additional considerations, such as scaling, for appropriate modeling.

Because of this connection of the log‐likelihood ratio function derived from the multivariate transformation model with Fisher's discriminant analysis, in the following we refer to disease classifiers based on log(L(y)) as transformation discriminant analysis (TDA).

### 3.3 Relationship to Logistic Regression

Logistic regression models the log‐likelihood ratio as a linear function of the biomarkers, $\log L\left(y\right)=\beta_{0}+\beta^{⊤}y$. Under Corollary 2, TDA with a shared covariance matrix has the same linear structure, but operates on the transformed biomarkers h(y) rather than the raw values. This generalizes logistic regression to arbitrary marginal distributions without altering the linear form of the score.

When disease‐specific covariance matrices $\Sigma_{0}\neq \Sigma_{1}$ are assumed, the log‐likelihood ratio from Proposition 1 acquires an additional quadratic term,

$$\log L\left(y\right)\propto -\frac{1}{2}\;h{\left(y\right)}^{⊤}\left(\Sigma_{1}^{-1}-\Sigma_{0}^{-1}\right)h\left(y\right),$$

which encodes pairwise interactions between the transformed biomarkers, weighted by the difference in their class‐specific precision matrices. Logistic regression cannot recover this term without explicitly constructing interaction features, whereas TDA obtains it directly from the joint model structure. An analogous mechanism operates under the location‐scale model of Proposition 2. The log‐likelihood ratio takes the quadratic form ${\left(h\left(y\right)-\beta \right)}^{⊤}A\left(h\left(y\right)-\beta \right)$, where $A={\Gamma \Sigma }_{1}^{-1}\Gamma -\Sigma_{0}^{-1}$. Under a shared covariance $\Sigma_{0}=\Sigma_{1}=\Sigma$, this reduces to $A={\Gamma \Sigma }^{-1}\Gamma -\Sigma^{-1}$, whose (j,k) off‐diagonal element equals ${\left(\Sigma^{-1}\right)}_{\mathrm{jk}}\left(\exp(-\gamma_{j}-\gamma_{k})-1\right)$. This is non‐zero whenever biomarkers j and k are correlated and at least one carries a non‐trivial scale shift, so the scale matrix Γ encodes pairwise biomarker interactions through the correlation structure even without disease‐specific covariance matrices.

### 3.4 Parameterization and Estimation

We parameterize the transformation function $h_{d}\left(y\right)=h_{d}\left(y|\theta_{d}\right)$ via parameters $\theta_{d}={\left(\vartheta_{d1}^{⊤},\ldots ,\vartheta_{\mathrm{dJ}}^{⊤}\right)}^{⊤}\in {\mathbb{R}}^{J\left(M+1\right)}$. The j th element $\vartheta_{\mathrm{dj}}$ of this vector parameterizes the marginal transformation function $h_{\mathrm{dj}}\left(y\right)=h_{\mathrm{dj}}\left(y|\vartheta_{\mathrm{dj}}\right)$ for the j th biomarker in either diseased (d=1) and nondiseased (d=0) classes. We describe the j th marginal transformation function in terms of a monotonically nondecreasing polynomial in Bernstein form

(4) $$h_{\mathrm{dj}}\left(y|\vartheta_{\mathrm{dj}}\right)=b_{j}{\left(y\right)}^{⊤}\vartheta_{\mathrm{dj}}=\sum_{m=0}^{M}\vartheta_{\mathrm{djm}}b_{\mathrm{jm}}\left(y\right)\;\text{for}\;y\in \mathbb{R},$$

where $b_{j}\left(y\right)={\left(b_{j0}\left(y\right),\ldots ,b_{\mathrm{jM}}\left(y\right)\right)}^{⊤}$ is a vector of M+1 basis functions with associated coefficients $\vartheta_{\mathrm{dj}}={\left(\vartheta_{\mathrm{dj}1},\ldots ,\vartheta_{\mathrm{dj}\left(M+1\right)}\right)}^{⊤}\in {\mathbb{R}}^{M+1}$ for j=1,…,J [32, 36]. The Bernstein basis polynomial of order M is defined on the interval [l,u] as

(5) $$b_{\mathrm{jm}}\left(y\right)=\left(\begin{array}{c} M\\ m \end{array}\right){\tilde{y}}^{m}{\left(1-\tilde{y}\right)}^{M-m},\;m=0,\ldots ,M,$$

where $\tilde{y}=\frac{y-l}{u-l}\in \left[0,1\right]$. The constraint $\vartheta_{\mathrm{djm}}\leq \vartheta_{\mathrm{dj}\left(m+1\right)}$ for m=0,…,M−1, guarantees the monotonicity of $h_{\mathrm{dj}}$ and the smooth parameterization ensures the existence of the derivative $h_{\mathrm{dj}}^{\prime }\left(y|\vartheta_{\mathrm{dj}}\right)=b_{j}^{\prime }{\left(y\right)}^{⊤}\vartheta_{\mathrm{dj}}$. Weierstrass' theorem ensures that any real‐valued continuous function can be approximated uniformly on the interval [l,u] with increasing order M [37]. With large enough orders M, the marginal distribution functions $\mathbb{P}\left(Y_{\mathrm{dj}}\leq y_{j}\right)=\Phi \left(b_{j}{\left(y_{j}\right)}^{⊤}\vartheta_{\mathrm{dj}}\right)$ closely approximate the marginal empirical cumulative distribution functions (ECDFs) used in the partially nonparametric estimation of nonparanormal models [28]. The benefit of compromising marginal fit by using moderate orders M is the ability to formulate and optimize the log‐likelihood simultaneously for all marginal parameters and the parameters defining the unstructured Gaussian copula. In practice, M does not require formal tuning. Choosing M larger than necessary increases computing time but does not harm estimation [38]. Orders M<10 fit most distributions encountered in practice reasonably well, and we use M=6 throughout.

We parameterize $\Sigma_{d}$ defining this copula in terms of the Cholesky factor of the precision matrix $\Sigma_{d}^{-1}={\tilde{\Lambda }}_{d}^{⊤}{\tilde{\Lambda }}_{d}$ and standardize the lower triangular J×J unit matrix $\Lambda_{d}$ such that ${\tilde{\Lambda }}_{d}=\Lambda_{d}\mathrm{diag}{\left(\Lambda_{d}^{-1}\Lambda_{d}^{-⊤}\right)}^{12}$ and thus $\mathrm{diag}\left(\Sigma_{d}\right)=1$. From a computational point of view, quadratic forms $h_{d}{\left(y|\theta_{d}\right)}^{⊤}\Sigma_{d}^{-1}h_{d}\left(y|\theta_{d}\right)$ and determinants $|\Sigma_{d}|$ arising in the log‐likelihood and log‐likelihood ratio functions simplify to $h_{d}{\left(y\right)}^{⊤}{\tilde{\Lambda }}_{d}^{⊤}{\tilde{\Lambda }}_{d}h_{d}\left(y\right)$ and $|\Sigma_{d}|\;={\left(\prod_{j=1}^{J}\mathrm{diag}{\left({\tilde{\Lambda }}_{d}\right)}_{j}\right)}^{-2}$, respectively. The term $\sqrt{\delta^{⊤}\Sigma^{-1}\delta }$ in [2] and [3] simplifies to $∥\tilde{\Lambda }\delta {∥}_{2}$ when $\Sigma_{d}=\Sigma$. Furthermore, the lower triangular elements $\lambda_{d}\in {\mathbb{R}}^{J\left(J-1\right)/2}$ of $\Lambda_{d}$ are unconstrained yet lead to a positive definite correlation matrix $\Sigma_{d}\left(\lambda_{d}\right)$.

The log‐likelihood contribution of a single observation y being an absolutely continuous vector of biomarker results, is given by

$$\ell \left(\theta_{d},\lambda_{d}|y\right)=\log\left(\phi_{0,\Sigma_{d}\left(\lambda_{d}\right)}\left(h_{d}\left(y|\theta_{d}\right)\right)\right)+\sum_{j=1}^{J}\log\left(h_{\mathrm{dj}}^{\prime }\left(y_{j}|\vartheta_{\mathrm{dj}}\right)\right)\;\text{for}\;d=0,1.$$

The maximum likelihood estimate of $\left(\theta_{d},\lambda_{d}\right)$ is derived from a sample of $N_{d}$ independent and identically distributed observations from either diseased (d=1) or nondiseased (d=0) subjects using constrained maximization algorithms. While the general parameterization and estimation procedure are detailed elsewhere [32], our application introduces a novel standardization of the Cholesky factors ${\tilde{\Lambda }}_{d}$. This standardization ensures that each j th transformation function can be interpreted as a marginal distribution function on the probit scale, which is essential for deriving model‐based AUCs and ROC curves. Disease‐specific score functions and theoretical properties of the likelihood‐based inference are described in [39].

Note that in the case of location‐scale marginal models, the transformation functions $h_{d}$ share parameters between both classes. Each marginal transformation function is then

(6) $$h_{\mathrm{dj}}\left(y|\vartheta_{j},\delta_{j},\gamma_{j}\right)=\frac{b_{j}{\left(y\right)}^{⊤}\vartheta_{j}-\delta_{j}d}{\exp\left(\gamma_{j}d\right)}$$

and one has to maximize the joint likelihood of both diseased and undiseased subjects with respect to the common parameters 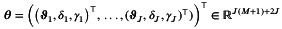 in addition to $\lambda_{0}$ and $\lambda_{1}$ (which one might want to be equal) based on all $N=N_{0}+N_{1}$ subjects.

### 3.5 Alternative Marginal Distributions

With F:ℝ→[0,1] denoting an absolutely continuous cumulative distribution function with a log‐concave density, the marginal distributions in our framework can be expressed as $\mathbb{P}\left(Y_{\mathrm{dj}}\leq y_{j}\right)=F\left(h_{\mathrm{dj}}\left(y_{j}\right)\right)$, where $h_{\mathrm{dj}}$ is a monotonically increasing transformation function. In the joint multivariate model this leads to the following transformation function

$$h_{d}\left(y|\theta_{d}\right)={\left(\Phi^{-1}\left(F\left(h_{d1}\left(y_{1}|\vartheta_{1}\right)\right)\right),\ldots ,\Phi^{-1}(F(h_{\mathrm{dJ}}(y_{J}|\vartheta_{J})))\right)}^{⊤}.$$

These choices are analogous to link functions in generalized linear models (GLMs). For example, using $F={\text{logit}}^{-1}$ leads to a log‐odds interpretation, while $F={\text{cloglog}}^{-1}$ corresponds to hazard‐based interpretations. Different selections of F lead to alternative marginal models and influence the interpretation of location and scale parameters. In our application, we use F=Φ (the standard normal CDF) for computational simplicity, but more robust alternatives such as $F={\text{logit}}^{-1}$ may be preferred in practice due to their interpretability and robustness properties [40].

### 3.6 Missing and Censored Biomarkers

Our proposed multivariate transformation model derives an optimal diagnostic score by combining multiple biomarkers through a likelihood ratio function. However, in practice, biomarker measurements may be partially missing due to feasibility constraints or only a subset may be available at training and test time. Our framework accommodates such cases by computing likelihood ratios using the marginal distribution of the observed biomarkers.

Without loss of generality, suppose biomarker $Y_{1}$ is missing at random and we observe the subset $y^{*}=\left(y_{2},\ldots ,y_{J}\right)$. By modeling the full joint distribution of $Y_{d}$ and applying the law of total probability, we obtain the marginal likelihood

$$f_{d}\left(y^{*}\right)=\phi_{0,\Sigma_{d}^{-1,-1}}\left(h_{d2}\left(y_{2}\right),\ldots ,h_{\mathrm{dJ}}\left(y_{J}\right)\right)\prod_{j=2}^{J}h_{\mathrm{dj}}^{\prime }\left(y_{j}\right),$$

where $\Sigma_{d}^{-1,-1}$ denotes the removal of the first row and column of $\Sigma_{d}$. This allows us to compute the likelihood ratio

$$L\left(y^{*}\right)=\frac{f_{1}\left(y_{2},\ldots ,y_{J}\right)}{f_{0}\left(y_{2},\ldots ,y_{J}\right)}$$

and classify subjects even when certain biomarkers are unobserved. Similarly, under the location‐scale simplification, one can compute the model‐based ROC curve and AUC using only the relevant subset of parameters $\delta_{d}^{-1}$ and $\Gamma_{d}^{-1}$.

More generally, the nonparanormal likelihood formulation allows direct incorporation of missingness into the estimation procedure [39]. Let $S_{i}\subseteq \left\{1,\ldots ,J\right\}$ denote the index set of observed biomarkers for subject i. The contribution of subject i to the incomplete‐data log‐likelihood is

$$\ell_{i}\left(\theta \right)=\log\phi_{0,\Sigma_{d}^{S_{i}}}\;\left(h_{d,S_{i}}\left(y_{i}^{*}\right)\right)+\sum_{j\in S_{i}}\log h_{\mathrm{dj}}^{\prime }\left(y_{\mathrm{ij}}\right),$$

where $\Sigma_{d}^{S_{i}}$ is the submatrix of $\Sigma_{d}$ indexed by $S_{i}$ and $h_{d,S_{i}}$ are the corresponding marginal transformation functions. The full incomplete‐data log‐likelihood $\ell \left(\theta \right)=\sum_{i=1}^{n}\ell_{i}\left(\theta \right)$ is then maximized over all parameters θ. Rather than discarding partially observed cases, our method uses the observed biomarker values for each subject, leading to potentially greater efficiency. The same framework extends naturally to biomarkers subject to lower or upper limits of detection, where the likelihood integrates over the censored regions. A detailed worked example for time‐varying prognostic biomarkers has been presented elsewhere [41].

### 3.7 High‐Dimensional Biomarker Panels

TDA is primarily designed for combining small to moderate numbers of biomarkers (J≲10), a regime common in clinical practice where each biomarker in the panel is known to carry diagnostic information. For moderately larger panels (10≲J≲20), the joint model remains applicable but begins to strain. This is because the Cholesky parameterization of $\Sigma_{d}$ requires J(J−1)/2 off‐diagonal parameters per class and the optimization problem becomes increasingly complex relative to the available sample size.

When J is large (J>20) and many biomarkers may be uninformative, we recommend a two‐stage procedure that preserves TDA's flexibility while controlling the effective dimensionality. In the first screening stage, a univariate marginal transformation model is fitted independently for each biomarker j=1,…,J. This gives the following log‐likelihood ratio contributions

$$m_{\mathrm{ij}}=\log f_{1}\left(y_{\mathrm{ij}}|{\hat{\vartheta }}_{j}\right)-\log f_{0}\left(y_{\mathrm{ij}}|{\hat{\vartheta }}_{j}\right),\;i=1,\ldots ,N.$$

These marginal contributions form an N×J feature matrix M, which is passed to a LASSO‐penalized logistic regression with penalty selected by cross‐validation [42]. The subset $\hat{S}=\left\{j:{\hat{\beta }}_{j}\neq 0\right\}$ of biomarkers with non‐zero coefficients at the one‐standard‐error penalty is then used in the second stage, where a full multivariate transformation model is fitted on $\hat{S}$ alone. By delegating variable selection to the LASSO, we inherit data‐adaptive regularization without introducing additional tuning parameters. The noise stability analysis in Section 4.5 shows that this two‐stage TDA procedure (TDA 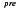) remains competitive with LASSO logistic regression at high noise dimensions while retaining TDA's ability to model nonlinear and asymmetric marginal distributions. The procedure admits the full range of marginal parameterizations and covariance structures described above, but in high‐dimensional settings parsimony is generally advisable. We find that location‐only marginal models with a shared covariance structure tend to be sufficient and lead to more stable estimation.

## 4 Empirical Evaluation

We assessed the performance of transformation discriminant analysis (TDA) for optimally combining multiple biomarkers for disease diagnosis using simulation studies. The aims were to: (i) evaluate performance under commonly assumed data generating processes; and (ii) assess the effects of model misspecification.

### 4.1 Methods Compared

We compared six variants of the proposed multivariate transformation model discussed in Section 3: disease‐specific marginal transformations (sTDA), location‐scale marginal transformations (lsTDA) and location‐only marginal transformations (TDA), each with either a global or disease‐specific correlation structure. The latter are denoted as sTDA 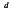, lsTDA 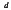, and TDA 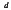.

As benchmarks, we included popular classification methods: logistic regression (LR), generalized additive models (GAM), random forests (RF) and eXtreme Gradient Boosting (XGBoost). We also evaluated classical discriminant analysis approaches: linear (LDA), quadratic (QDA), mixture (MDA), and flexible discriminant analysis (FDA). Additionally, we implemented linear combination methods proposed in the biomarker literature, including the step‐down approach [7] (KT) and the min‐max method [5] (LIU). For reference, we also computed the AUC corresponding to the true likelihood ratio.

### 4.2 Simulation Setup and Scenarios

We considered four biomarkers to match the HCC application in Section 5 and simulated data under five distinct scenarios. Each scenario was evaluated at total sample sizes N∈{50,100,200}. To assess the impact of class imbalance, we considered disease prevalences of 10% and 50%. These sample sizes reflect those commonly encountered in medical studies focused on developing or validating biomarker combinations. We generated 1000 replications per scenario, with an independent large test dataset of size 20 000 with 50% prevalence for out‐of‐sample evaluation. Performance was assessed using out‐of‐sample (OOS) AUC. Additional metrics, including bias and standard errors of the AUC are reported in the Appendix.

#### 4.2.1 Scenario A: Multivariate Normal Biomarkers

This scenario represents the ideal setting for classical LDA and logistic regression. We generated data from multivariate normal distributions with equal covariance matrices for diseased and nondiseased groups. Means were $\mu_{0}={\left(0,0,0,0\right)}^{⊤}$ for nondiseased and $\mu_{1}={\left(-0.2,0.3,0.7,-0.1\right)}^{⊤}$ for diseased subjects. These shifts were chosen to yield an approximate true AUC of 0.8. The common covariance matrix $\Sigma =\Sigma_{0}=\Sigma_{1}$ was estimated from the HCC biomarker data

$$\Sigma =\left(\begin{array}{cccc} 1.00 & 0.17 & 0.36 & 0.32\\ 0.17 & 1.00 & 0.41 & 0.45\\ 0.36 & 0.41 & 1.00 & 0.82\\ 0.32 & 0.45 & 0.82 & 1.00 \end{array}\right)$$

#### 4.2.2 Scenario B: Multivariate Skewed Biomarkers

To evaluate robustness to skewed distributions, we considered two variants.

- Multivariate log‐normal distributions with log‐scale means matched to Scenario A.
- Biomarkers generated from the following skewed distributions:

Nondiseased: $\left(N\left(0.6,1\right),\;\chi^{2}\left(2.5\right),\;\mathrm{Exp}\left(1\right),\;\Gamma \left(1.2,1\right)\right)$.

Diseased: $\left(N\left(1.1,1\right),\;\chi^{2}\left(3\right),\;\mathrm{Exp}\left(1.7\right),\;\Gamma \left(2,1\right)\right)$

Here, $\chi^{2}\left(k\right)$ represents the chi‐squared distribution with k degrees of freedom, Exp(λ) is the exponential distribution with rate λ, and Γ(α,β) represents the gamma distribution with shape parameter α and rate parameter β. Covariance matrices were the same as in Scenario A.

#### 4.2.3 Scenario C: Disease‐Specific Dependence

To reflect disease‐specific dependence structures between biomarkers, we allowed different correlation matrices in the two disease groups. Marginals were kept identical to Scenarios A or B.

Correlation matrices were estimated from the HCC dataset:

$$\Sigma_{0}=\left(\begin{array}{cccc} 1.00 & 0.05 & 0.24 & 0.10\\ 0.05 & 1.00 & 0.23 & 0.35\\ 0.24 & 0.23 & 1.00 & 0.62\\ 0.10 & 0.35 & 0.62 & 1.00 \end{array}\right)\;\Sigma_{1}=\left(\begin{array}{cccc} 1.00 & 0.17 & 0.33 & 0.31\\ 0.17 & 1.00 & 0.41 & 0.40\\ 0.33 & 0.41 & 1.00 & 0.92\\ 0.31 & 0.40 & 0.92 & 1.00 \end{array}\right)$$

Such differences in structure are known to reduce the performance of methods assuming homogeneous dependence such as logistic regression [12].

#### 4.2.4 Scenario D: Tail Dependence

To investigate the impact of dependence misspecification, we generated data from the Clayton (strong lower tail dependence) and Gumbel copulas (upper tail dependence). Copula parameters were estimated from the HCC application dataset (Clayton: θ=0.4146; Gumbel: θ=1.3170).

#### 4.2.5 Scenario E: Logistic Model

In this setting, data were generated directly from logistic regression models, for which discriminative approaches are well suited and the TDA modeling assumptions are violated. We evaluated two settings:

- Linear model: log odds of disease as a linear function of Y, that is, $\text{logit}\left(\mathbb{P}\left(D=1|Y\right)=\beta_{0}+\beta^{⊤}Y\right)$, where $Y∼N\left(0,1_{4}\right)$, $\beta_{0}=0.5$ and β=(0.5,−0.6,1.1,0.4) was chosen to yield a true AUC of approximately 0.80.
- Interaction model: log odds of disease including pairwise interactions between biomarkers.

$$\begin{array}{cc} \text{logit}\left(\mathbb{P}\left(D=1|Y\right)\right) & =-0.5+1.2Y_{1}-0.8Y_{2}+0.6Y_{3}^{2}-0.4Y_{4}^{2}\\ & \;+0.7Y_{1}Y_{2}-0.5Y_{3}Y_{4}. \end{array}$$

In both cases, the class label D was generated via Bernoulli sampling from the implied probability. This scenario tests the robustness to model misspecification for generative methods like TDA when the true model aligns with a discriminative paradigm.

### 4.3 Results

**FIGURE 1:**
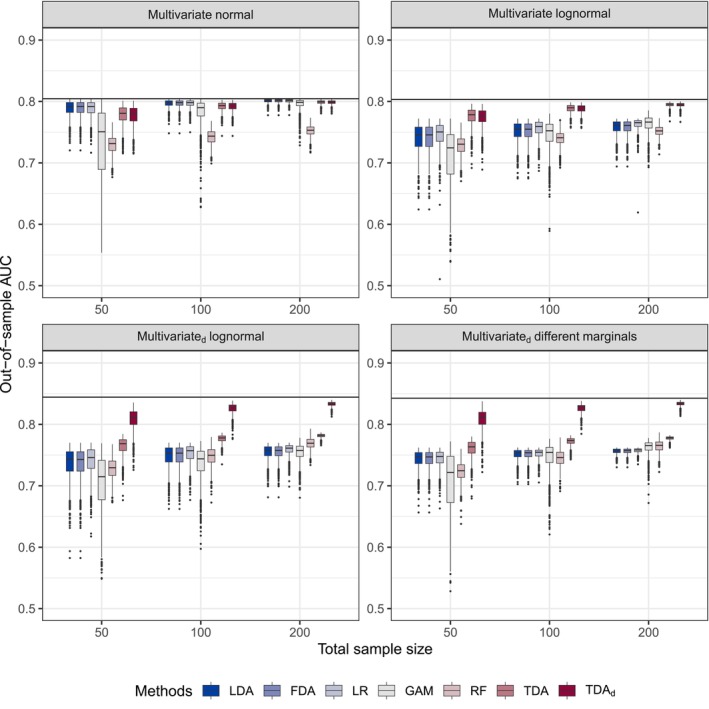
Empirical out‐of‐sample area under the receiver operating characteristic curve (AUC) across different simulation settings (Scenarios A, B and C) for a 50% prevalence scenario. Box‐plots are color‐coded to categorize methods, with our proposed approaches represented by TDA. The optimal AUC in each setting is marked by the black line.

**FIGURE A1:**
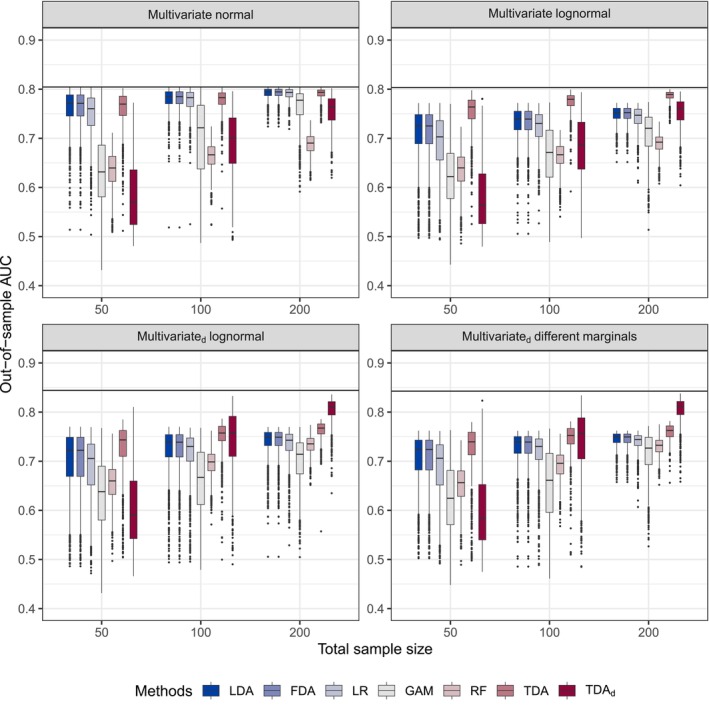
Empirical out‐of‐sample area under the receiver operating characteristic curve (AUC) across different simulation settings (Scenarios A, B and C) for a 10% prevalence scenario. Box‐plots are color‐coded to categorize methods, with our proposed approaches represented by TDA. The optimal AUC in each setting is marked by the black line.

We summarized the results of the empirical OOS AUCs under Scenario A, B and C in the case of 50% prevalence in Figure 1. For the main text, we present results for the TDA and TDA 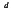 models, which we consider as default parameterizations. For comparison, we use LDA, FDA, LR, GAM and RF as the most commonly applied alternatives. A similar plot for 10% prevalence can be found in (Appendix Figure A1). Complete results for bias and variance of the OOS AUC for all methods are available in the (Appendix Table A1).

#### 4.3.1 Scenarios A‐B: Gaussian and Skewed Marginals

When biomarkers followed a multivariate normal distribution, all methods performed similarly and approached the optimal AUC with increasing sample sizes. However, under skewed marginals (log‐normal or different marginal distributions), performance differed. LR and LDA declined due to violated normality assumptions. GAM and RF showed instability, particularly at small sample sizes.

In contrast, TDA models remained stable and accurate across all marginal distributions. Their parametric flexibility and faster convergence resulted in low‐variance and reasonable AUCs even in small samples. However, the more complex disease‐specific TDA variants (TDA 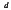, lsTDA 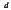, sTDA 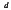) showed slightly higher bias and increased variance in low prevalence settings, especially where modeling separate covariance structures offered limited benefit.

#### 4.3.2 Scenario C: Disease‐Specific Dependence

Methods assuming a shared correlation structure suffered when disease‐specific dependence was present, especially methods like LR, similar to prior findings [12]. Disease‐specific TDA models (TDA 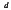, TDA 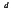, lsTDA 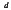) performed well, whilst models without disease‐specific dependence had more bias. LR and GAM, often perceived as assumption‐agnostic, underperformed. This highlights that they implicitly assume equal dependence structures across disease populations.

#### 4.3.3 Scenario D: Tail Dependence

None of the methods fully captured tail dependence. Tree‐based methods (RF, XGBoost) struggled the most, exhibiting high variance and bias. Among the TDA variants, location‐only and location‐scale versions (TDA, lsTDA) were most robust, showing moderate bias and consistent performance. This suggests that TDA's parametric structure tolerates modest misspecification better than black‐box alternatives, especially at small sample sizes.

#### 4.3.4 Scenario E: Logistic Model

In the correctly specified linear logistic model, LR and LDA unsurprisingly had the lowest bias. TDA followed closely. The sTDA and lsTDA variants exhibited slightly higher bias due to additional parameters but remained more stable than flexible competitors like GAM, RF, and XGBoost. In the interaction setting, all methods experienced performance drops, with LR and LDA affected most due to model misspecification. TDA variants showed reasonable robustness, particularly lsTDA and lsTDA 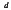, which maintained lower variance and modest bias. As discussed in Section 3, both the location‐scale parameterisation and disease‐specific covariance structure implicitly encode pairwise interactions between transformed biomarkers through the quadratic form of the log‐likelihood ratio. These interactions cannot be captured by a linear score such as logistic regression without explicit feature construction. This structural flexibility partially compensates for the interaction terms in the true model, explaining the relative robustness of these variants even under misspecification.

### 4.4 Robustness to Missing Data

To assess TDA's behavior under incomplete training data, we conducted an additional simulation under Scenario A (N=200, 50% prevalence) with missing completely at random (MCAR) values introduced independently in two of the four biomarkers at proportions of 30%, 50% and 70%. Missingness was limited to the training set and all methods were evaluated on the same complete test set. We compared a missing‐aware TDA, which uses incomplete training data in the likelihood, against complete‐case (CC) alternatives for TDA, LR and RF, each trained after deletion. Oracle models trained on fully complete data served as upper bounds.

**FIGURE A2:**
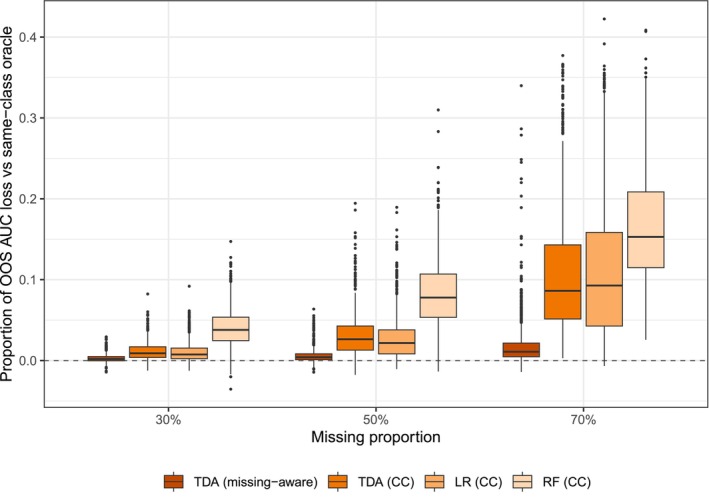
Proportion of out‐of‐sample AUC loss relative to a same‐class oracle trained on complete data, for increasing missingness proportions in two of four biomarkers (Scenario A: Multivariate normal copula, shifted normal marginals; N=200, 50% prevalence). TDA (missing‐aware) uses the incomplete‐data likelihood and all complete‐case (CC) methods discard observations with missing values prior to training.

Results are summarized in Appendix (Figure A2). The missing‐aware TDA incurred negligible AUC loss relative to its oracle across all missingness rates, demonstrating that the incomplete‐data likelihood effectively recovers the information discarded by complete case methods. Complete‐case methods also proved surprisingly robust under Scenario A. Even at 50% missingness, median proportion of AUC loss remained below 5% for both TDA (CC) and LR (CC). We expect the advantage of the missing‐aware approach to be more pronounced in settings with skewed marginals or disease‐specific dependence structures, where the distributional information lost through deletion could be harder to recover from the remaining complete cases.

### 4.5 Higher Dimensions and Robustness to Noise Variables

To assess TDA's model stability under high‐dimensional uninformative inputs, we conducted a simulation across two scenarios (N=200, 50% prevalence): multivariate normal biomarkers with a shared covariance matrix (Scenario A) and mixed skewed marginals with disease‐specific covariance (Scenarios B and C). Training and test data were augmented with k independent standard normal noise variables, for k∈{1,…,20,30,50,75,100,200,500,1000}. We compared all six TDA variants up to k=20, and TDA 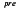, LR 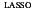, and RF across all k, recording OOS AUC, log‐likelihood, and runtime over 50 replications.

**FIGURE 2:**
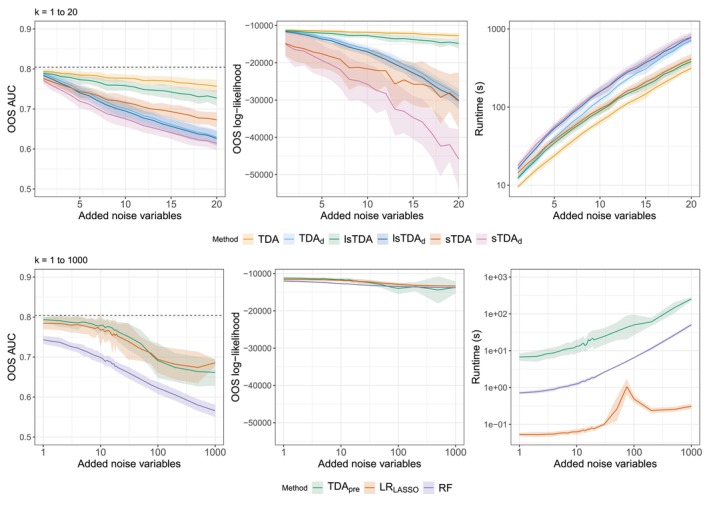
Noise stability under increasing numbers of added uninformative variables (k) for multivariate normal biomarkers with shared covariance (Scenario A). Top row: All six TDA variants up to k=20. Bottom row: TDA 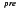 (LASSO‐based pre‐screening), LR 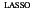, and RF over the full noise sequence (k on log scale). Left: Out‐of‐sample AUC, with the dashed line indicating the oracle. Middle: Out‐of‐sample Bernoulli log‐likelihood. Right: Runtime in seconds (log scale). Lines and ribbons represent means ± one standard deviation over 50 replications.

**FIGURE A3:**
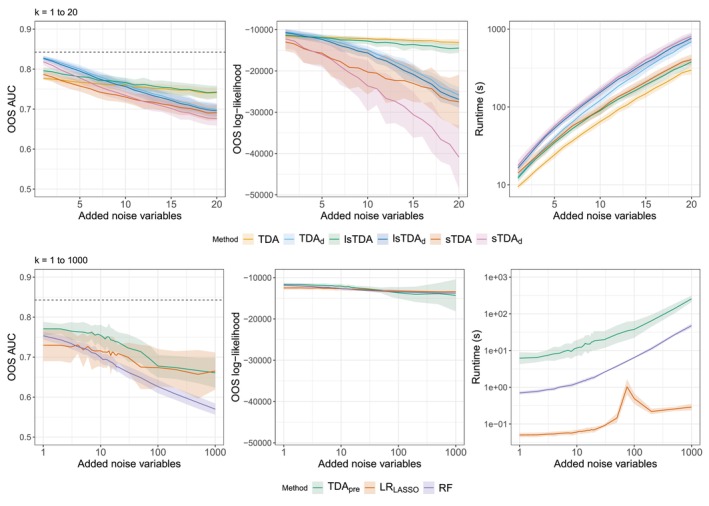
Noise stability under increasing numbers of added uninformative variables (k) for mixed skewed marginals with disease‐specific covariance (Scenarios B and C). Layout as in Figure 2.

Results are summarized in Figure 2 (Scenario A) and Appendix Figure A3 (Scenarios B and C). As expected, all methods degrade as k increases, with AUC and log‐likelihood declining and runtimes growing. Among the six TDA variants, the location‐only model (TDA) showed the most robustness, degrading slowly as k increased, consistent with its parsimonious parameterization. More complex variants, in particular those with disease‐specific covariance structures (TDA 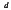, lsTDA 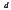, sTDA 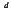), exhibited faster performance degradation and substantially longer runtimes as k grew. Under Scenario A, where the DGP is well‐suited to logistic regression, TDA 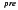 and LR 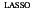 performed similarly across the full noise sequence. Under the more complex DGP of Scenarios B and C, TDA 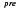 offered some gains. However, at very high noise levels, the simpler LR 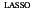 is a reasonable and a computationally cheaper alternative.

## 5 Optimal Diagnostic Test for Hepatocellular Carcinoma

### 5.1 Serum Biomarker Data and Multivariate Model

We analyzed published data from a case–control study involving N=401 subjects, consisting of $N_{1}=208$ subjects with hepatocellular carcinoma (HCC) and nondiseased group of $N_{0}=193$ subjects diagnosed with liver cirrhosis, all of whom exhibited viral or non‐viral etiology [17, 43]. The diagnosis of HCC and liver cirrhosis was established through histological examinations. Imaging studies were conducted on patients with liver cirrhosis to exclude hepatocellular carcinoma.

Plasma samples from these subjects were analyzed for multiple biomarkers, including alpha‐fetoprotein (AFP), protein induced by vitamin K absence or antagonist‐II (PIVKA‐II), osteopontin (OPN), and Dickkopf‐1 (DKK‐1). While AFP stands as the most established biomarker for HCC diagnosis, its standalone diagnostic performance can fall short [44]. The goal of our analysis was to create an optimal diagnostic test using a combination of the measured biomarkers for the detection of HCC and thereby capturing different aspects of HCC heterogeneity.

**FIGURE A4:**
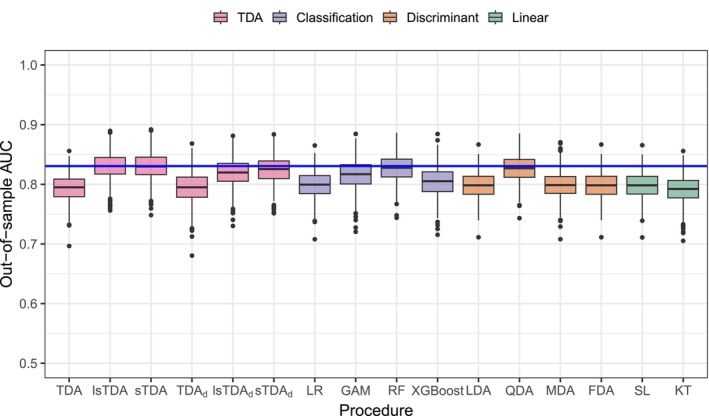
Distributions of out‐of‐sample (OOS) area under the curve (AUC) values obtained by combining four biomarkers (AFP, PIVKA‐II, OPN, and DKK‐1) to generate an optimal diagnostic score. The box‐plots are color‐coded to categorize methods with those using the multivariate transformation model (TDA), and competitors including binary classification, discriminant analysis and linear combination methods. The blue line indicates the maximum median OOS AUC which was for the lsTDA method.

Given the right‐skewed distributions of all markers, we initially performed a logarithmic transformation of their measurement values. Unlike our TDA approach or tree‐based (random forest and boosting) methods, other candidate methods are not invariant to such monotone transformations and will benefit from more symmetric marginal biomarker distributions. We assessed the various complexities of our methods and compared them with competitor methods, detailed in Section 4. We used a repeated holdout validation procedure with a 50–50 data splits over 1000 replications to estimate the out‐of‐sample (OOS) AUC and compare competing methods. The resulting empirical out‐of‐sample AUCs are shown in (Appendix E Figure A4). The multivariate transformation model with location‐scale marginal models and a global covariance matrix (lsTDA) yielded the highest median empirical out‐of‐sample AUC, leading us to select it for the subsequent analysis.

Table 1 displays the coefficient estimates from the lsTDA multivariate transformation model for the HCC biomarkers (DKK‐1, OPN, PIVKA‐II, AFP) along with their corresponding 95% confidence intervals. Intervals were computed by a parametric bootstrap with 1000 replications, where each replicate was obtained by sampling model parameters from a multivariate normal distribution with mean equal to the maximum likelihood estimates and covariance matrix equal to the variance–covariance matrix of the fitted model. The estimated marginal transformation functions for each of the biomarkers are provided in Section F Figure A10 of the Appendix. Positive location terms signify that individuals with HCC exhibit higher biomarker values compared to those without HCC, with the magnitude indicating the strength of the location shift. Positive scale terms suggest that biomarker values for HCC subjects display greater variability than those without HCC. This pattern holds true for all biomarkers, indicating consistently elevated biomarker measurements and increased variability in subjects with HCC.

**TABLE 1:** Estimated coefficients of the multivariate transformation model with location‐scale marginals and global correlation matrix (lsTDA), along with their corresponding 95% confidence intervals (CI), for the biomarkers employed in hepatocellular carcinoma diagnosis.

Variable	Coefficient (95% CI)
Location $\delta_{j}$	
DKK‐1	0.721 (0.471, 0.935)
OPN	0.780 (0.443, 1.059)
PIVKA‐II	1.257 (0.982, 1.527)
AFP	1.572 (1.262, 1.859)
Scale $\gamma_{j}$	
DKK‐1	0.499 (0.232, 0.762)
OPN	1.232 (0.987, 1.553)
PIVKA‐II	0.694 (0.444, 0.974)
AFP	0.753 (0.495, 1.060)
Correlation Σ	
OPN—DKK‐1	0.104 (0.011, 0.187)
PIVKA‐II—DKK‐1	0.302 (0.210, 0.375)
PIVKA‐II—OPN	0.315 (0.227, 0.404)
AFP—DKK‐1	0.232 (0.141, 0.310)
AFP—OPN	0.348 (0.256, 0.421)
AFP—PIVKA‐II	0.833 (0.789, 0.861)

### 5.2 Model‐Based ROC Curves and AUC

**FIGURE 3:**
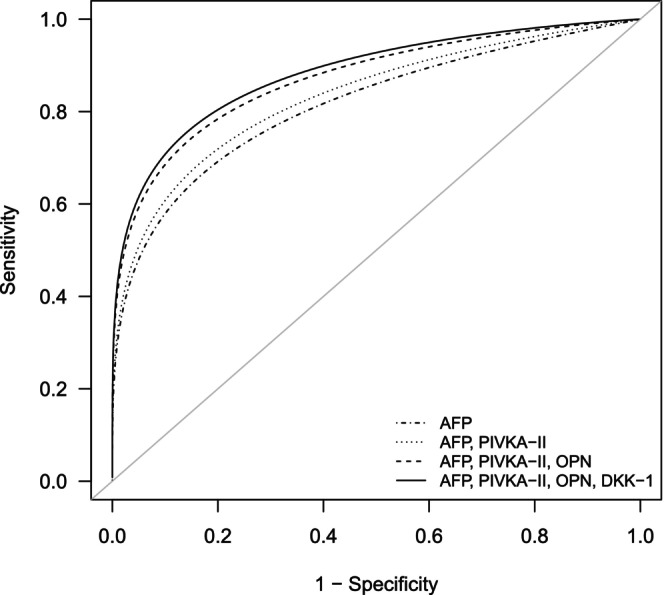
Estimated model‐based ROC curves for the cumulative diagnostic benefit of adding biomarkers to AFP for hepatocellular carcinoma diagnosis.

Figure 3 displays the estimated optimal model‐based ROC curves obtained from likelihood ratio combinations of biomarker subsets. AFP was placed first, as it is the most commonly used marker in diagnostic studies of HCC. Additional biomarkers were added sequentially in order of increasing marginal AUC. While this ordering was arbitrary, Table 2 presents results for all possible permutations to provide a comprehensive evaluation. Each subset yields a distinct optimal ROC curve, and as expected, diagnostic accuracy generally improves with the inclusion of additional biomarkers. Note that all combinations can be evaluated from the same fitted model, enabling efficient assessment of multiple diagnostic strategies without refitting. The optimal ROC curve for the full biomarker combination, together with 95% confidence bands, is shown in Appendix Figure A9.

**TABLE 2:** Estimated optimal model‐based and out‐of‐sample AUCs for the likelihood ratio combination of biomarkers, along with their corresponding 95% confidence intervals (CI).

Combination	AUC (95% CI)	Mean OOS AUC (95% CI)
AFP	0.814 (0.773, 0.850)	0.775 (0.728, 0.823)
PIVKA‐II	0.767 (0.721, 0.809)	0.721 (0.671, 0.775)
OPN	0.716 (0.678, 0.753)	0.690 (0.636, 0.742)
DKK‐1	0.674 (0.628, 0.719)	0.656 (0.606, 0.707)
AFP & PIVKA‐II	0.832 (0.793, 0.866)	0.782 (0.731, 0.827)
AFP & OPN	0.858 (0.825, 0.887)	0.815 (0.766, 0.858)
AFP & DKK‐1	0.833 (0.797, 0.870)	0.801 (0.756, 0.849)
AFP & PIVKA‐II & OPN	0.871 (0.840, 0.898)	0.820 (0.777, 0.864)
AFP & PIVKA‐II & DKK‐1	0.849 (0.815, 0.883)	0.805 (0.760, 0.849)
AFP & OPN & PIVKA‐II	0.871 (0.840, 0.898)	0.821 (0.776, 0.865)
AFP & OPN & DKK‐1	0.872 (0.841, 0.900)	0.826 (0.784, 0.867)
AFP & DKK‐1 & PIVKA‐II	0.849 (0.815, 0.883)	0.806 (0.761, 0.846)
AFP & DKK‐1 & OPN	0.872 (0.841, 0.900)	0.826 (0.783, 0.867)
AFP & PIVKA‐II & OPN & DKK‐1	0.883 (0.855, 0.910)	0.832 (0.789, 0.873)

Table 2 reports both model‐based AUCs with 95% confidence intervals and the corresponding mean out‐of‐sample (OOS) AUCs. AFP alone had a model‐based AUC of 0.814 (95% CI: 0.769–0.850) and an OOS AUC of 0.775. The full four‐biomarker model achieved the highest performance, with a model‐based AUC of 0.883 (95% CI: 0.854–0.912) and a mean OOS AUC of 0.831, highlighting the value of combining markers. Among three‐biomarker combinations, those including AFP, OPN, and either DKK‐1 or PIVKA‐II achieved nearly comparable model‐based AUCs of 0.872 and relatively high OOS AUCs of 0.826. This demonstrates that meaningful gains can be made even without using all four biomarkers.

**FIGURE 4:**
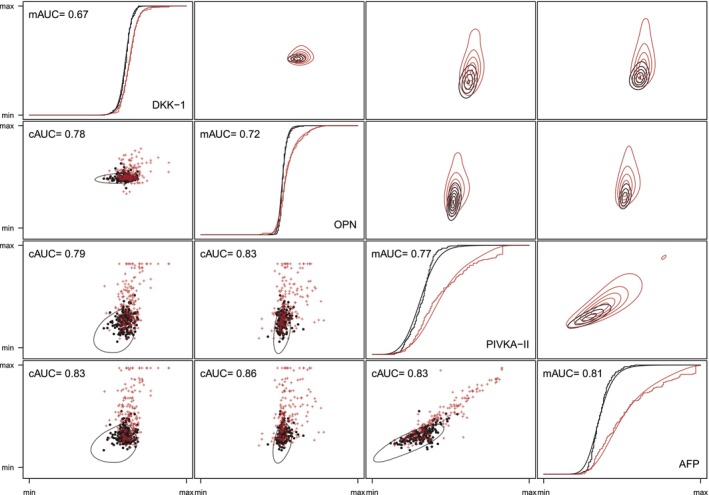
Visualization of a multivariate transformation model with location‐scale marginals and global correlation matrix (lsTDA). Diagonal: empirical (step) and modeled (smooth) marginal distribution functions for each biomarker. The marginal model‐based AUC (mAUC) for diagnosing HCC using each individual biomarker is provided in the top left of each panel. Lower off‐diagonal: bivariate scatterplot of biomarker combinations with subjects without HCC (denoted by points ⋅) and with HCC (denoted by +). The gray line represents the modeled likelihood ratio function decision boundary. The cumulative model‐based AUC (cAUC) for each optimal bivariate biomarker combination is provided in the top left of each panel. Upper off‐diagonal: estimated bivariate density function for each biomarker combination. In all plots, black denotes no HCC, and red signifies HCC.

We also explored all pairwise combinations, as depicted in Figure 4. On the diagonal of this figure, the marginal CDFs estimated using polynomials in Bernstein form (with M=6) approximate the marginal ECDFs well. However, the marginal models for the HCC subjects do not fit particularly well for PIVKA‐II and AFP due to some extreme measurements. These observations may be due to upper detection limits for the biomarkers, which would need to be appropriately addressed in the model by right‐censoring, but were beyond the scope of this analysis.

The lower off‐diagonal plots feature two‐dimensional scatterplots of the biomarker data. Recall that the likelihood ratio combination of biomarkers classifies a subject as an HCC case if their composite score exceeds some cutoff c (here log(L(y))>0 was used), signifying stronger evidence for an HCC diagnosis. The gray line marks the decision boundary of the modeled likelihood ratio function in the two‐dimensional marker space under this rule. The most effective bivariate combination involves OPN and AFP, nearly reaching the same cAUC as using all four markers.

### 5.3 Covariate Dependent Analysis

**FIGURE 5:**
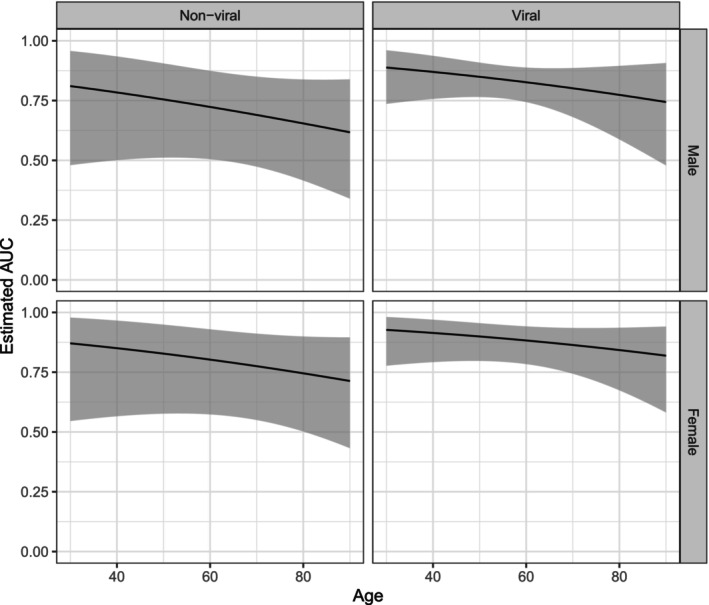
Estimated covariate‐dependent AUCs using the composite OOS likelihood ratio score of a multivariate transformation model with location‐scale marginals and global correlation matrix (lsTDA), segmented by age and etiological groups, distinguishing between viral causes (HBV, HCV) and other etiologies such as alcohol‐related or cryptogenic factors.

The initial analysis of the data found that covariates such as age, gender, and HCC etiology influenced the individual diagnostic performance of biomarkers [17] observed that covariates such as age, gender and HCC etiology influenced the individual diagnostic performance of biomarkers. To evaluate how these factors affect the diagnostic accuracy of the composite score, we employed a covariate‐specific AUC model defined by

$$\mathrm{AUC}\left(x\right)=\frac{\exp\left(\delta \left(x\right)\right)\left(\exp(\delta (x))-1-\delta \left(x\right)\right)}{{\left(\exp\left(\delta \left(x\right)\right)-1\right)}^{2}},$$

where x represents the covariates age, gender, and etiology, and δ(x) denotes the covariate effect on the ROC curve. Briefly, AUC(x) arises from a univariate TDA model with $F={\text{logit}}^{-1}$ featuring a covariate‐dependent location term δ(x), further details are available in [40]. To ensure unbiased AUCs, we initially computed an average out‐of‐sample log‐likelihood ratio score and then examined the covariates' dependence on this score. The results are depicted in Figure 5. For comparison, we used a random forest to generate a similar score based on the conditional class probability, yielding results consistent with our method (Figure A11). The composite score shows higher diagnostic accuracy for younger ages with gender not having a substantial impact. Furthermore, our composite score improves the accuracy of HCC detection for viral etiologies, despite the documented lower accuracy of AFP in identifying viral‐related HCC [45, 46]. This improvement is likely a result of the complementary information from other markers within the likelihood ratio combination.

## 6 Discussion

Accurate diagnostic tests are essential for routine surveillance and timely identification of diseases. In this article, we proposed a multivariate modeling framework called transformation discriminant analysis (TDA) to combine multiple biomarkers using the likelihood ratio function. TDA offers flexibility and allows modeling of key clinical complexities such as skewed marginals, disease‐specific dependence structures and missing biomarkers. Its parametric form enables likelihood‐based inference for model parameters and diagnostic accuracy metrics.

Across a range of simulation scenario and ts, TDA consistently provided accurate diagnostic scores. In settings with skewed marginals or disease‐specific dependence structures, commonly used methods such as logistic regression and discriminant analysis showed notable degradation in performance. Machine learning approaches like random forests and GAMs also struggled, especially in small sample sizes, exhibiting high variability and slower convergence. In contrast, TDA models demonstrated lower variance and better adaptability to distributional shifts. In disease‐specific dependence scenarios, the advantage of correctly specifying correlation structures was evident. Even under model misspecification, such as tail‐dependent copula structures or when the true model followed a logistic regression, TDA variants remained competitive.

**FIGURE A7:**
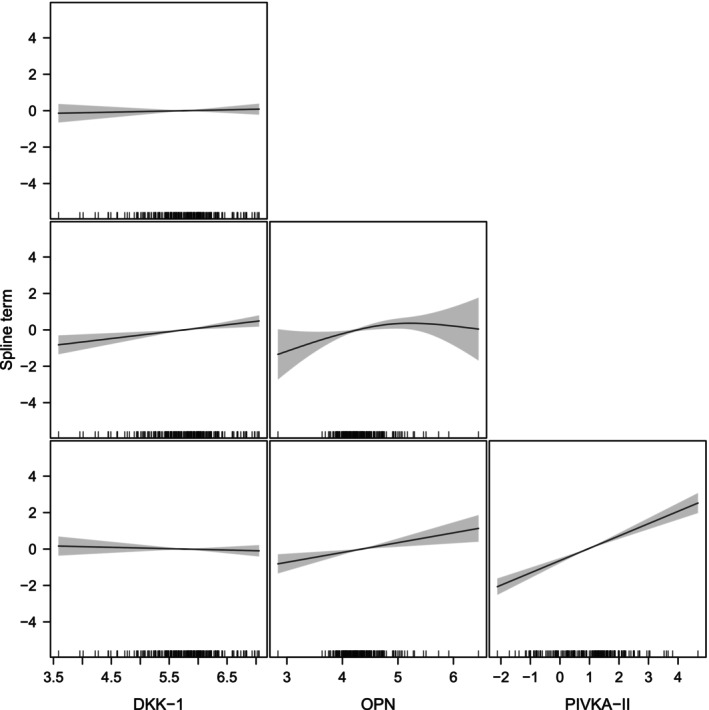
Estimated transformation functions $g_{\mathrm{jk}}$ for 2≤k<j≤4 as a function of the conditioned biomarker values in subjects without hepatocellular carcinoma.

**FIGURE A8:**
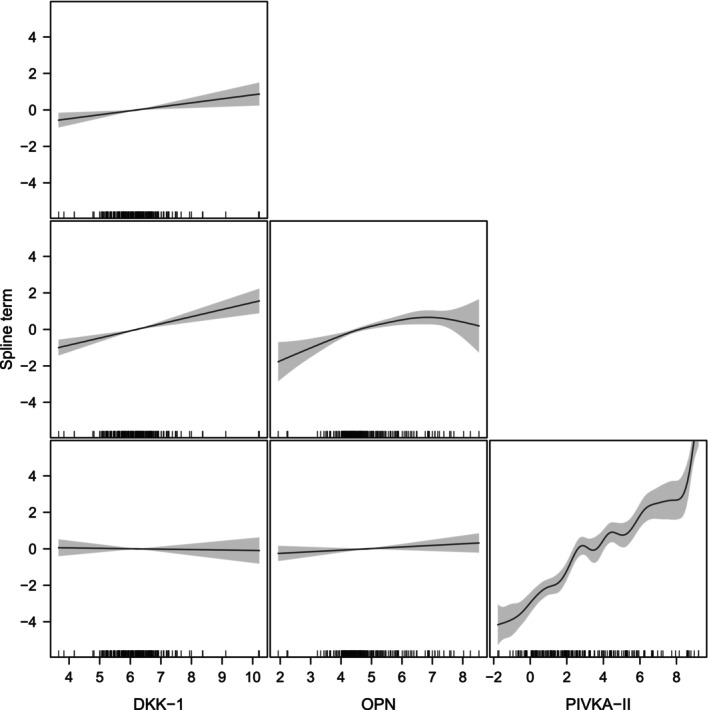
Estimated transformation functions $g_{\mathrm{jk}}$ for 2≤k<j≤4 as a function of the conditioned biomarker values in subjects with hepatocellular carcinoma.

**FIGURE A9:**
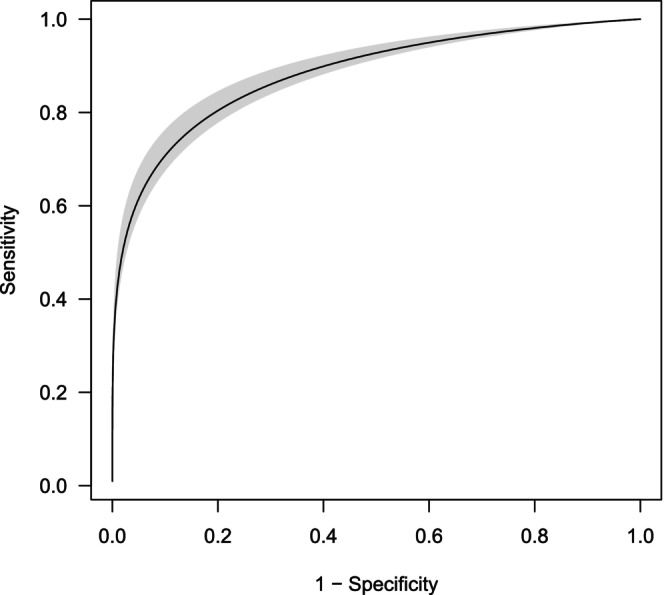
Estimated model‐based ROC curves and pointwise 95% confidence band for the optimal combination of AFP, PIVKA‐II, OPN and DKK.

**FIGURE A10:**
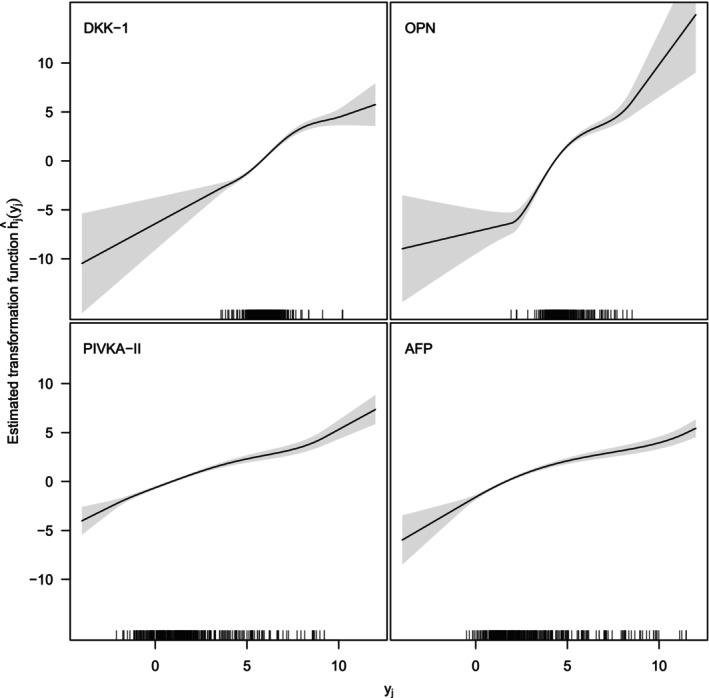
Estimated transformation functions for the multivariate transformation model with location‐scale marginals and global correlation matrix (lsTDA) of hepatocellular carcinoma biomarkers.

**FIGURE A11:**
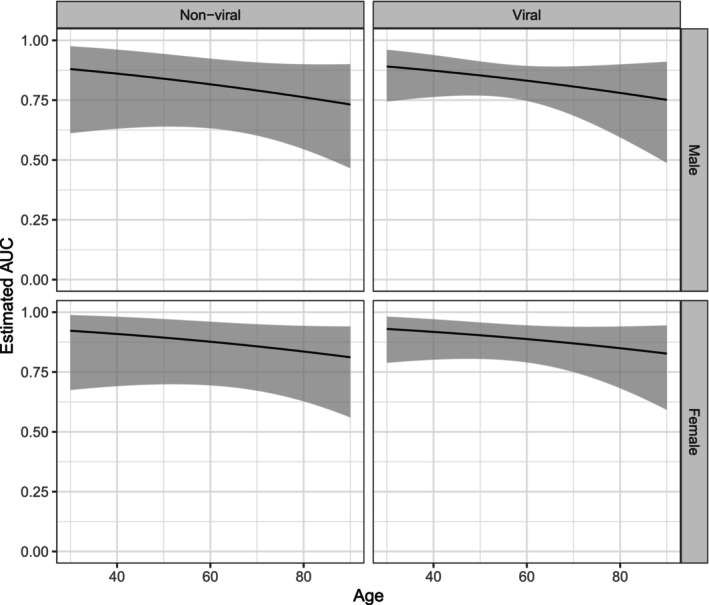
Estimated covariate‐dependent AUCs for the conditional class probabilities from the random forest, segmented by age and etiological groups, distinguishing between viral causes (HBV, HCV) and other etiologies such as alcohol‐related or cryptogenic factors.

When modeling the dependence structure of multivariate data using a parametric copula family, such as the Gaussian copula used in our approach, a common challenge lies in the potential misspecification of the copula family. While we explored the severity of this issue through simulations with tail‐dependent copulas, our evaluation remains confined to parametric settings. In this context, if any of the conditional regressions lacks monotonicity, the reliability of a copula model for describing the joint distribution diminishes [47]. Fortunately, this assumption can be empirically verified by plotting estimated conditional transformations, as done in Figures A7 and A8, which revealed largely monotonic behavior.

We further assessed model fit using the multivariate probability integral transform [48]. The results (Appendix E.2) indicated good overall fit, though minor issues were detected in modeling AFP values near the upper limit of detection. These could be addressed by treating such observations as right‐censored or adapting more flexible marginal models. Further, selecting a biomarker panel in practice is rarely a question of diagnostic accuracy alone. Resource constraints means that a clinician may prefer a smaller, cheaper combination that achieves most of the accuracy of the full panel. TDA naturally supports this kind of cost‐constrained panel selection. Practitioners can enumerate candidate subsets, evaluate their model‐based AUCs directly and identify the combination that best balances accuracy against resource use. We formalize this as an integer optimization problem in Appendix C.

Additional simulations further characterized TDA. Under class imbalance (10% prevalence), TDA retained its advantages over competing methods, though all methods exhibited higher variance at small sample sizes, consistent with the reduced effective number of disease cases. Under MCAR data, a missing‐aware TDA that incorporates incomplete observations directly into the likelihood incurred negligible AUC loss relative to the oracle, while complete‐case alternatives remained surprisingly robust. The noise stability simulations showed that the simple parameterizations were the most resilient to added uninformative variables. More complex variants with disease‐specific covariance structures degraded faster and ran considerably slower as dimensionality grew. For larger biomarker panels, the proposed two‐stage prescreening strategy recovered performance by restricting the joint model to variables selected by LASSO.

More broadly, high‐dimensional settings with many more biomarkers than subjects (J≫N) remain challenging for the current framework. In such settings, the covariance matrices required by TDA are not full rank, which can impair estimation. A potential extension to TDA involves fitting flexible univariate transformation models, mapping observations to an approximately normal scale and applying penalized covariance estimation techniques—similar in spirit to the nonparanormal model [28]. Because the penalty appears symmetrically in the likelihood of the diseased and nondiseased populations, it cancels out in the likelihood ratio. However, this approach warrants further investigation to assess its validity and practical performance in high‐dimensional applications.

While TDA provides a powerful framework for biomarker combinations, it is not a replacement for all existing methods. Discriminative approaches like logistic regression or methods which optimize empirical performance metrics may outperform in scenarios where the likelihood ratio is difficult to estimate accurately or where our model‐based assumptions do not hold. Instead, TDA complements these methods by offering a generative perspective with interpretable components, theoretical optimality guarantees and built‐in mechanisms for model assessment. Unlike black‐box machine learning models, TDA facilitates inspection of estimated distributions and transformations. This can help practitioners understand the contribution of individual biomarkers and evaluate modeling assumptions. This transparency can guide further model refinement and inform clinical decisions.

A reference implementation of transformation discriminant analysis is available in the tram add‐on package [49] to the R system for statistical computing. The empirical results presented in Sections 4 and 5 can be reproduced by.

install.packages("tram")
library("tram")
demo("hcc", package = "tram")




## Funding

This work was supported by Schweizerischer Nationalfonds zur Förderung der Wissenschaftlichen Forschung, 200021_219384.

## Conflicts of Interest

The authors declare no conflicts of interest.

## Figures and Tables

**FIGURE A5:**
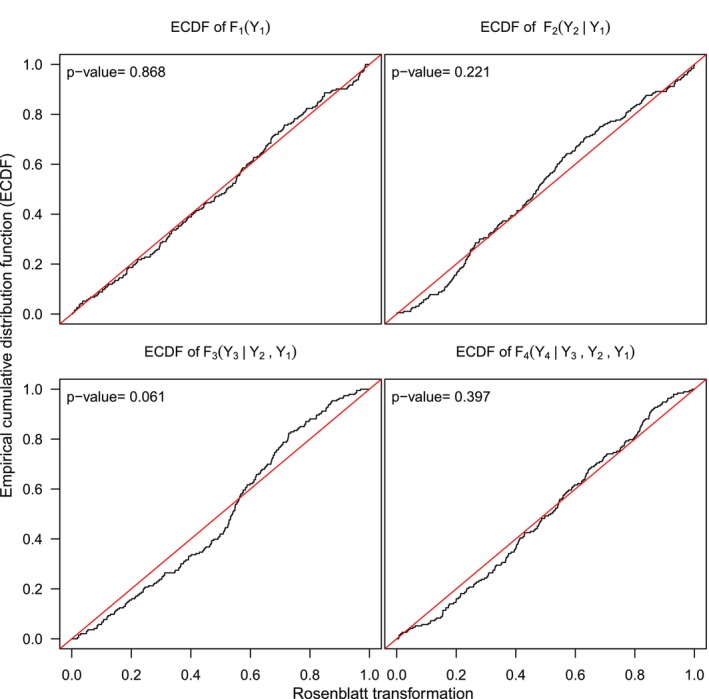
Model diagnostic plots for the multivariate transformation model with location‐scale marginals and global correlation matrix (lsTDA) in subjects without hepatocellular carcinoma, employing the Rosenblatt transformation. The black line represents the empirical cumulative distribution function (ECDF) of the marginal Rosenblatt transformed data, while the red line represents the theoretical CDF of a uniform distribution.

**FIGURE A6:**
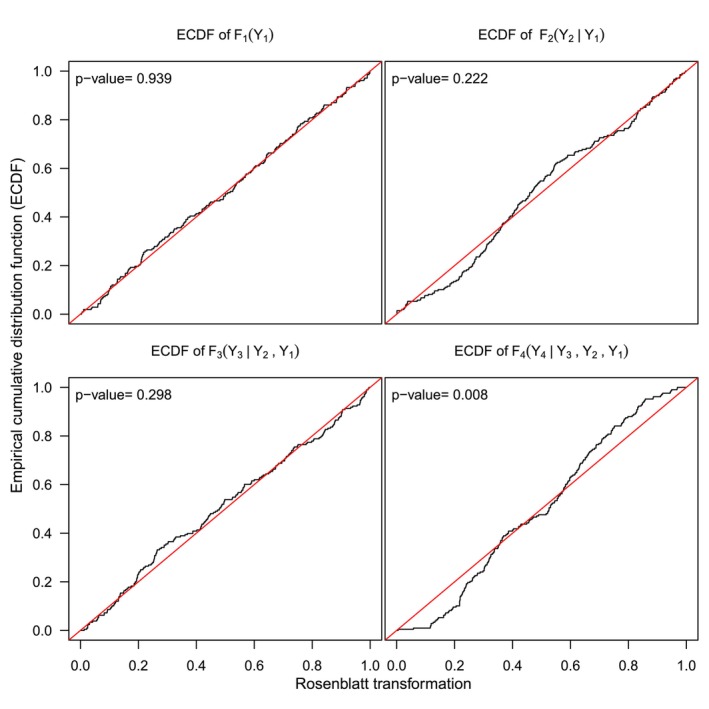
Model diagnostic plots for the multivariate transformation model with location‐scale marginals and global correlation matrix (lsTDA) in subjects with hepatocellular carcinoma, employing the Rosenblatt transformation. The black line represents the empirical cumulative distribution function (ECDF) of the marginal Rosenblatt transformed data, while the red line represents the theoretical CDF of a uniform distribution.

## Data Availability

The data that support the findings of this study are openly available in Dryad at https://doi.org/10.5061/dryad.3n901, reference number 44697.

## Appendix A Computational Details

We used **mvtnorm** [50] version 1.3‐7 to sample from multivariate normal distributions, **pracma** [51] version 2.4.6 for numerical integration of ROC curves, and **qrng** [52] version 0.0‐11 for random number generation. Furthermore, we used **tramME** [53] version 1.0.8 for estimating conditional distributions, **mgcv** [54] version 1.9‐4 for evaluating the generalized chi‐square distribution, and **copula** [55] version 1.1‐7 for simulating from copulas with tail‐dependence.

In evaluating competitor methods, we used **randomForest** [56] version 4.7‐1.2 with 5000 trees, each with a minimum of 10 observations in terminal nodes and fitted gradient boosting models with **xgboost** [57] version 3.2.1.1 where the iterations were selected by 5‐fold cross‐validation. For linear and quadratic discriminant analysis, we used **MASS** [58] version 7.3‐65 and mixture and flexible discriminant analysis were performed using **mda** [59] version 0.5‐5.

All computations were performed using R version 4.5.3 [60].

## Appendix B Proofs

We restate and present the proofs of our propositions and their corollaries in this section. Proposition 1 gives the log‐likelihood ratio function which has fully flexible marginal distributions whilst Proposition 2 introduces a location‐scale simplification of the marginal distributions which leads to distributional results for the scalar composite score, $L_{d}=L\left(Y_{d}\right)$ for d={0,1} in Corollary 1. Equivalence of a special case of our model with the results of [3] is shown in Corollary 2. Proof of Proposition 1 The modeled density function $f_{d}$ is the multivariate normal density function with a zero mean vector and a correlation matrix $\Sigma_{d}$, evaluated at the transformed variables $h_{d}\left(y\right)$, and incorporates the product of the derivatives of the transformation functions. Its logarithm is given by

$$\log\left(f_{d}\left(y\right)\right)=-\frac{1}{2}\left(h_{d}{\left(y\right)}^{⊤}\Sigma_{d}^{-1}h_{d}\left(y\right)+J\log\left(2\pi \right)+\log(|\Sigma_{d}|)\right)+\sum_{j=1}^{J}\log\left(h_{\mathrm{dj}}^{\prime }\left(y_{j}\right)\right).$$

The asserted representation follows from substituting into the definition of the log‐likelihood ratio.

$$\log\left(L\left(y\right)\right)=\log\left(f_{1}\left(y\right)\right)-\log\left(f_{0}\left(y\right)\right).$$

Proof of Proposition 2 Using Proposition 1 with $h_{0}\left(y\right)=h\left(y\right)$ and $h_{1}\left(y\right)=\Gamma \left(h\left(y\right)-\delta \right)$ we have

with constant $c=-\frac{1}{2}\left(\log\left(\frac{\left|\Sigma_{1}\right|}{\left|\Sigma_{0}\right|}\right)-\delta^{⊤}\Sigma_{0}^{-1}\left(I+{\left(\Sigma_{0}A\right)}^{-1}\right)\delta \right)-\sum_{j=1}^{J}\gamma_{j}.$

Definition 1 Let $X_{1},\ldots ,X_{m}$ be independent normally distributed variables with $X_{i}∼N\left(\mu_{i},1\right)$. The distribution of $\sum_{i=1}^{m}w_{i}X_{i}^{2}$ is called the generalized chi‐square distribution with parameter vectors specifying the weights $w={\left(w_{1},\ldots ,w_{m}\right)}^{⊤}$, degrees of freedom $k=1\in {\mathbb{R}}^{m}$ and non‐centrality terms $\nu ={\left(\mu_{1}^{2},\ldots ,\mu_{m}^{2}\right)}^{⊤}$.
Lemma 1
*(Quadratic form of the multivariate normal distribution). Let* $L=X^{⊤}\mathrm{AX}$ *with* $X∼N_{J}\left(\mu ,\Sigma \right)$, rank(A)=J *and* Σ *symmetric and positive semi‐definite. Let the spectral decomposition of* $\Sigma^{\frac{1}{2}}A\Sigma^{\frac{1}{2}}$ *be given by* ${\mathrm{PWP}}^{⊤}$. *Then* L *has a generalized chi‐square distribution with weights which are the eigenvalues* w=diag(W), *degrees of freedom* $1\in {\mathbb{R}}^{J}$ *and non‐centrality parameters* $\mathrm{diag}{\left(P^{⊤}\Sigma^{\frac{1}{2}}\mu \right)}^{2}$.
Proof of Lemma 1 Using the spectral decomposition we can rewrite L as

where $Q=P^{⊤}\Sigma^{-\frac{1}{2}}X∼N_{J}\left(P^{⊤}\Sigma^{-\frac{1}{2}}\mu ,I\right)$. Thus, by definition, L has a generalized chi‐square distribution with the given parameters.
Corollary 1
*Let the spectral decomposition of* ${\tilde{\Sigma }}_{d}^{\frac{1}{2}}A{\tilde{\Sigma }}_{d}^{\frac{1}{2}}$ *be given by* $P_{d}W_{d}P_{d}^{⊤}$ *where* ${\tilde{\Sigma }}_{d}$ *is the correlation matrix of* $h\left(Y_{d}\right)$. *Then the scalar composite score* $L_{d}$ *follows a generalized chi‐square distribution* $G_{\chi_{J}^{2}}\left(w_{d},\nu_{d}\right)$ *with weights as* $w_{d}=\mathrm{diag}\left(W_{d}\right)$, *the non‐centrality parameters* $\nu_{d}=\mathrm{diag}{\left(P_{d}^{⊤}\Sigma_{d}^{-\frac{1}{2}}\left(\delta_{d}-\beta \right)\right)}^{2}$ *and the degrees of freedom* $1\in {\mathbb{R}}^{J}$.
Proof of Corollary 1 From Proposition 2 we have that

and the distributions of the transformed random vectors in the two classes are

$$h\left(Y_{0}\right)∼N_{J}\left(0,\Sigma_{0}\right)\;\text{and}\;h\left(Y_{1}\right)∼N_{J}\left(\delta ,\Gamma^{-1}\Sigma_{1}\Gamma^{-1}\right).$$

The result follows from an application of Lemma 1 with $X=h\left(Y_{d}\right)-\beta$.

## Appendix C Cost and Resource Optimization

Interest may also lie in finding a balance between cost‐effectiveness and diagnostic accuracy. We can use the property of the multivariate model detailed in Section 3.6 to formulate this task as an integer optimization problem. Define the decision variables $s={\left(s_{1},\ldots ,s_{J}\right)}^{⊤}$ where $s_{j}=0$ or 1, indicating if a biomarker is rejected or accepted in the overall biomarker combination. Assume K resources (e.g., machines, cost or time) where $a_{\mathrm{kj}}$ is the amount of resource k used on biomarker j and $b_{k}$ is the budget for the k th resource. To find the optimal assignment $s^{*}$, we solve the optimization problem

$$\begin{array}{ll} {\mathrm{maximize}}_{s}\mathrm{AUC}\left(s\right) & =\Phi \left(\sqrt{\frac{{\left(⊙s\right)}^{⊤}\left(\mathrm{diag}\left(s\right){\hat{\Sigma }}^{-1}\mathrm{diag}\left(s\right)\right)\left(\hat{\delta }⊙s\right)}{2}}\right)\\ & \text{subject to}\sum_{j=1}^{J}a_{\mathrm{kj}}s_{j}\leq b_{k}\;\left(k=1,\ldots ,K\right),\\ & s_{j}=0\;\text{or}\;1\;\left(j=1,\ldots ,J\right). \end{array}$$

Here, ⊙ denotes the Hadamard product (element‐wise product), and $\hat{\delta }$ and $\hat{\Sigma }$ are the estimated model coefficients from a location‐only model as presented in the model‐based AUC function. The objective is to maximize the diagnostic accuracy of the biomarker combination without exceeding the limited availability of any resource $b_{k}$. The optimization problem can be solved without needing to re‐fit joint distributions for biomarker subsets. Note that for more complex models outlined previously, the objective function can be similarly formulated. While these AUC functions might not be readily available in closed form, they can be computed numerically based on the model parameters.

## Appendix D Empirical Evaluation Additional Results

**TABLE A1:** Bias and standard error (in parentheses) of out‐of‐sample AUC estimates for different methods. Results are stratified by sample size, prevalence and simulation scenarios. Values represent averages over 1000 simulation replicates.

	Total sample size					
	50% prevalence			10% prevalence		
	50	100	200	50	100	200
Scenario A—multivariate normal						
TDA	0.026 (0.015)	0.012 (0.007)	0.006 (0.003)	0.045 (0.037)	0.027 (0.021)	0.014 (0.011)
TDA	0.028 (0.016)	0.013 (0.007)	0.006 (0.003)	0.218 (0.070)	0.113 (0.063)	0.049 (0.032)
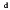
lsTDA	0.034 (0.017)	0.016 (0.008)	0.007 (0.004)	0.132 (0.071)	0.071 (0.044)	0.033 (0.022)
lsTDA	0.034 (0.018)	0.016 (0.008)	0.007 (0.004)	0.226 (0.066)	0.132 (0.061)	0.063 (0.036)
sTDA	0.053 (0.020)	0.031 (0.011)	0.017 (0.006)	0.194 (0.056)	0.144 (0.051)	0.085 (0.035)
sTDA	0.053 (0.020)	0.031 (0.011)	0.017 (0.006)	0.230 (0.051)	0.188 (0.056)	0.109 (0.040)
LDA	0.016 (0.013)	0.008 (0.006)	0.004 (0.003)	0.044 (0.040)	0.025 (0.023)	0.013 (0.012)
QDA	0.016 (0.013)	0.008 (0.006)	0.004 (0.003)	0.214 (0.069)	0.127 (0.060)	0.061 (0.036)
MDA	0.043 (0.029)	0.020 (0.013)	0.008 (0.005)	0.116 (0.059)	0.074 (0.038)	0.042 (0.021)
FDA	0.016 (0.013)	0.008 (0.006)	0.004 (0.003)	0.044 (0.040)	0.025 (0.023)	0.013 (0.012)
LR	0.016 (0.013)	0.008 (0.006)	0.004 (0.003)	0.057 (0.048)	0.028 (0.024)	0.014 (0.013)
GAM	0.072 (0.058)	0.022 (0.023)	0.009 (0.010)	0.170 (0.073)	0.103 (0.076)	0.040 (0.038)
RF	0.075 (0.015)	0.062 (0.012)	0.052 (0.009)	0.169 (0.039)	0.141 (0.028)	0.116 (0.021)
XGBoost	0.143 (0.030)	0.120 (0.024)	0.097 (0.019)	0.170 (0.045)	0.145 (0.032)	0.126 (0.025)
KT	0.082 (0.033)	0.076 (0.023)	0.070 (0.016)	0.123 (0.066)	0.097 (0.054)	0.082 (0.042)
LIU	0.202 (0.013)	0.198 (0.008)	0.195 (0.004)	0.217 (0.025)	0.215 (0.024)	0.208 (0.019)
Scenario B—multivariate different marginals						
TDA	0.024 (0.013)	0.014 (0.006)	0.010 (0.003)	0.052 (0.040)	0.030 (0.021)	0.018 (0.011)
TDA	0.026 (0.014)	0.014 (0.006)	0.010 (0.003)	0.212 (0.068)	0.112 (0.058)	0.054 (0.032)
lsTDA	0.029 (0.015)	0.014 (0.007)	0.007 (0.003)	0.123 (0.065)	0.064 (0.041)	0.031 (0.021)
lsTDA	0.030 (0.015)	0.014 (0.007)	0.007 (0.003)	0.217 (0.064)	0.118 (0.056)	0.060 (0.032)
sTDA	0.055 (0.020)	0.034 (0.012)	0.019 (0.007)	0.159 (0.063)	0.115 (0.049)	0.072 (0.031)
sTDA	0.055 (0.021)	0.034 (0.012)	0.019 (0.007)	0.199 (0.059)	0.154 (0.056)	0.096 (0.038)
LDA	0.029 (0.013)	0.022 (0.007)	0.018 (0.004)	0.066 (0.049)	0.043 (0.024)	0.031 (0.014)
QDA	0.047 (0.013)	0.040 (0.008)	0.037 (0.005)	0.209 (0.060)	0.135 (0.057)	0.084 (0.036)
MDA	0.069 (0.029)	0.048 (0.018)	0.036 (0.010)	0.149 (0.058)	0.109 (0.041)	0.078 (0.029)
FDA	0.029 (0.013)	0.022 (0.007)	0.018 (0.004)	0.066 (0.049)	0.043 (0.024)	0.031 (0.014)
LR	0.028 (0.013)	0.021 (0.007)	0.017 (0.003)	0.076 (0.053)	0.046 (0.028)	0.030 (0.016)
GAM	0.086 (0.059)	0.040 (0.030)	0.022 (0.014)	0.175 (0.070)	0.112 (0.073)	0.055 (0.041)
RF	0.059 (0.013)	0.051 (0.011)	0.044 (0.008)	0.144 (0.038)	0.122 (0.030)	0.101 (0.021)
XGBoost	0.120 (0.032)	0.100 (0.022)	0.081 (0.016)	0.147 (0.044)	0.131 (0.035)	0.112 (0.025)
KT	0.049 (0.023)	0.040 (0.016)	0.037 (0.012)	0.105 (0.067)	0.074 (0.046)	0.053 (0.031)
LIU	0.213 (0.011)	0.210 (0.007)	0.209 (0.004)	0.233 (0.026)	0.229 (0.025)	0.221 (0.021)
Scenario C—multivariate normal						
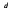
TDA	0.079 (0.013)	0.068 (0.007)	0.063 (0.003)	0.116 (0.048)	0.098 (0.037)	0.082 (0.017)
TDA	0.032 (0.016)	0.014 (0.007)	0.007 (0.003)	0.240 (0.077)	0.102 (0.064)	0.035 (0.024)
lsTDA	0.064 (0.018)	0.050 (0.011)	0.043 (0.007)	0.157 (0.077)	0.088 (0.046)	0.049 (0.021)
lsTDA	0.034 (0.016)	0.015 (0.008)	0.007 (0.003)	0.253 (0.070)	0.121 (0.064)	0.047 (0.027)
sTDA	0.085 (0.021)	0.064 (0.014)	0.052 (0.009)	0.240 (0.060)	0.174 (0.059)	0.101 (0.035)
sTDA	0.051 (0.018)	0.027 (0.010)	0.014 (0.005)	0.275 (0.047)	0.204 (0.065)	0.097 (0.039)
LDA	0.072 (0.012)	0.065 (0.006)	0.061 (0.003)	0.115 (0.050)	0.097 (0.038)	0.081 (0.017)
QDA	0.014 (0.009)	0.007 (0.005)	0.003 (0.002)	0.244 (0.072)	0.116 (0.062)	0.045 (0.027)
MDA	0.085 (0.030)	0.059 (0.019)	0.048 (0.014)	0.132 (0.058)	0.096 (0.042)	0.062 (0.027)
FDA	0.072 (0.012)	0.065 (0.006)	0.061 (0.003)	0.115 (0.050)	0.097 (0.038)	0.081 (0.017)
LR	0.073 (0.012)	0.065 (0.006)	0.062 (0.003)	0.125 (0.054)	0.097 (0.039)	0.078 (0.017)
GAM	0.124 (0.055)	0.079 (0.023)	0.066 (0.009)	0.204 (0.069)	0.161 (0.071)	0.104 (0.039)
RF	0.116 (0.016)	0.096 (0.013)	0.076 (0.009)	0.190 (0.039)	0.150 (0.031)	0.112 (0.019)
XGBoost	0.182 (0.032)	0.154 (0.028)	0.119 (0.021)	0.205 (0.047)	0.175 (0.038)	0.141 (0.026)
KT	0.119 (0.029)	0.110 (0.021)	0.105 (0.015)	0.168 (0.063)	0.146 (0.053)	0.120 (0.035)
LIU	0.181 (0.012)	0.177 (0.006)	0.175 (0.003)	0.197 (0.034)	0.189 (0.026)	0.183 (0.016)
Scenario C—multivariate lognormal						
TDA	0.079 (0.014)	0.068 (0.006)	0.063 (0.003)	0.118 (0.054)	0.093 (0.032)	0.080 (0.017)
TDA	0.036 (0.017)	0.019 (0.008)	0.011 (0.004)	0.238 (0.077)	0.102 (0.063)	0.041 (0.026)
lsTDA	0.063 (0.019)	0.047 (0.011)	0.040 (0.006)	0.154 (0.077)	0.081 (0.042)	0.049 (0.022)
lsTDA	0.038 (0.016)	0.019 (0.008)	0.012 (0.004)	0.250 (0.068)	0.119 (0.063)	0.053 (0.028)
sTDA	0.083 (0.022)	0.061 (0.013)	0.049 (0.008)	0.247 (0.068)	0.168 (0.066)	0.101 (0.039)
sTDA	0.055 (0.020)	0.031 (0.011)	0.018 (0.005)	0.282 (0.048)	0.206 (0.073)	0.101 (0.043)
LDA	0.108 (0.026)	0.096 (0.018)	0.090 (0.013)	0.144 (0.063)	0.122 (0.049)	0.105 (0.031)
QDA	0.100 (0.025)	0.093 (0.020)	0.087 (0.016)	0.280 (0.054)	0.218 (0.072)	0.161 (0.054)
MDA	0.145 (0.035)	0.127 (0.025)	0.113 (0.019)	0.204 (0.065)	0.174 (0.055)	0.147 (0.039)
FDA	0.108 (0.026)	0.096 (0.018)	0.090 (0.013)	0.144 (0.063)	0.122 (0.049)	0.105 (0.031)
LR	0.104 (0.024)	0.092 (0.015)	0.086 (0.010)	0.159 (0.065)	0.131 (0.052)	0.111 (0.034)
GAM	0.140 (0.047)	0.107 (0.027)	0.090 (0.014)	0.211 (0.068)	0.184 (0.067)	0.143 (0.049)
RF	0.116 (0.017)	0.097 (0.015)	0.076 (0.009)	0.190 (0.039)	0.149 (0.028)	0.111 (0.019)
XGBoost	0.184 (0.035)	0.156 (0.030)	0.120 (0.022)	0.206 (0.046)	0.175 (0.038)	0.142 (0.027)
KT	0.130 (0.028)	0.120 (0.021)	0.116 (0.017)	0.184 (0.060)	0.158 (0.050)	0.140 (0.038)
LIU	0.194 (0.011)	0.190 (0.005)	0.188 (0.001)	0.203 (0.031)	0.198 (0.025)	0.192 (0.013)
Scenario C—multivariate different marginals						
TDA	0.084 (0.014)	0.073 (0.007)	0.068 (0.003)	0.144 (0.058)	0.113 (0.040)	0.093 (0.025)
TDA	0.034 (0.015)	0.018 (0.007)	0.011 (0.003)	0.245 (0.078)	0.106 (0.066)	0.036 (0.023)
lsTDA	0.060 (0.016)	0.047 (0.010)	0.040 (0.006)	0.168 (0.081)	0.088 (0.051)	0.047 (0.022)
lsTDA	0.035 (0.015)	0.018 (0.007)	0.009 (0.003)	0.255 (0.071)	0.119 (0.066)	0.044 (0.025)
sTDA	0.087 (0.020)	0.065 (0.014)	0.051 (0.008)	0.225 (0.069)	0.163 (0.058)	0.099 (0.036)
sTDA	0.058 (0.018)	0.033 (0.011)	0.019 (0.006)	0.258 (0.061)	0.184 (0.067)	0.089 (0.037)
LDA	0.087 (0.014)	0.079 (0.007)	0.076 (0.005)	0.145 (0.057)	0.116 (0.038)	0.097 (0.021)
QDA	0.066 (0.011)	0.058 (0.007)	0.054 (0.004)	0.261 (0.064)	0.164 (0.068)	0.095 (0.036)
MDA	0.117 (0.030)	0.096 (0.019)	0.084 (0.012)	0.187 (0.062)	0.154 (0.047)	0.123 (0.031)
FDA	0.087 (0.014)	0.079 (0.007)	0.076 (0.005)	0.145 (0.057)	0.116 (0.038)	0.097 (0.021)
LR	0.087 (0.013)	0.079 (0.007)	0.075 (0.004)	0.153 (0.058)	0.117 (0.036)	0.097 (0.021)
GAM	0.148 (0.057)	0.102 (0.033)	0.081 (0.014)	0.233 (0.064)	0.187 (0.069)	0.128 (0.046)
RF	0.109 (0.014)	0.096 (0.012)	0.082 (0.009)	0.182 (0.040)	0.147 (0.029)	0.115 (0.018)
XGBoost	0.169 (0.033)	0.147 (0.024)	0.121 (0.018)	0.199 (0.048)	0.173 (0.036)	0.145 (0.024)
KT	0.101 (0.022)	0.091 (0.014)	0.087 (0.010)	0.165 (0.067)	0.128 (0.046)	0.105 (0.029)
LIU	0.251 (0.012)	0.248 (0.007)	0.245 (0.003)	0.272 (0.029)	0.267 (0.028)	0.258 (0.023)
Scenario D—Gumbel copula dependence						
TDA	0.036 (0.016)	0.026 (0.009)	0.021 (0.005)	0.073 (0.049)	0.048 (0.029)	0.032 (0.013)
TDA	0.038 (0.016)	0.027 (0.009)	0.023 (0.005)	0.224 (0.074)	0.132 (0.061)	0.071 (0.035)
lsTDA	0.038 (0.019)	0.021 (0.010)	0.013 (0.005)	0.139 (0.073)	0.080 (0.048)	0.043 (0.026)
lsTDA	0.038 (0.019)	0.021 (0.010)	0.013 (0.005)	0.229 (0.069)	0.139 (0.060)	0.075 (0.036)
sTDA	0.063 (0.024)	0.040 (0.014)	0.026 (0.008)	0.171 (0.067)	0.128 (0.053)	0.083 (0.034)
sTDA	0.064 (0.024)	0.040 (0.014)	0.026 (0.008)	0.209 (0.063)	0.166 (0.058)	0.105 (0.038)
LDA	0.032 (0.016)	0.022 (0.009)	0.018 (0.005)	0.077 (0.052)	0.052 (0.031)	0.037 (0.015)
QDA	0.047 (0.015)	0.038 (0.008)	0.034 (0.005)	0.221 (0.066)	0.142 (0.059)	0.087 (0.038)
MDA	0.070 (0.032)	0.043 (0.019)	0.030 (0.010)	0.166 (0.062)	0.121 (0.047)	0.085 (0.031)
FDA	0.032 (0.016)	0.022 (0.009)	0.018 (0.005)	0.077 (0.052)	0.052 (0.031)	0.037 (0.015)
LR	0.030 (0.014)	0.020 (0.007)	0.016 (0.004)	0.077 (0.061)	0.043 (0.032)	0.026 (0.013)
GAM	0.092 (0.062)	0.041 (0.033)	0.023 (0.015)	0.182 (0.082)	0.119 (0.083)	0.056 (0.042)
RF	0.061 (0.015)	0.051 (0.012)	0.042 (0.008)	0.166 (0.044)	0.135 (0.036)	0.106 (0.022)
XGBoost	0.122 (0.034)	0.097 (0.024)	0.077 (0.016)	0.168 (0.056)	0.143 (0.041)	0.114 (0.026)
KT	0.054 (0.024)	0.042 (0.013)	0.036 (0.007)	0.127 (0.073)	0.093 (0.057)	0.064 (0.035)
LIU	0.224 (0.009)	0.222 (0.007)	0.219 (0.004)	0.239 (0.025)	0.236 (0.023)	0.231 (0.019)
Scenario D—Clayton copula dependence						
TDA	0.030 (0.013)	0.020 (0.006)	0.015 (0.003)	0.073 (0.052)	0.044 (0.028)	0.028 (0.013)
TDA	0.030 (0.014)	0.018 (0.006)	0.014 (0.003)	0.206 (0.065)	0.128 (0.056)	0.069 (0.035)
lsTDA	0.037 (0.016)	0.022 (0.008)	0.015 (0.004)	0.137 (0.064)	0.082 (0.045)	0.046 (0.024)
lsTDA	0.037 (0.016)	0.022 (0.008)	0.015 (0.004)	0.211 (0.061)	0.134 (0.053)	0.077 (0.033)
sTDA	0.063 (0.019)	0.042 (0.013)	0.028 (0.008)	0.163 (0.065)	0.126 (0.052)	0.082 (0.031)
sTDA	0.063 (0.019)	0.042 (0.013)	0.028 (0.008)	0.194 (0.060)	0.155 (0.054)	0.103 (0.036)
LDA	0.044 (0.014)	0.036 (0.007)	0.032 (0.004)	0.095 (0.056)	0.066 (0.032)	0.050 (0.015)
QDA	0.068 (0.013)	0.061 (0.008)	0.058 (0.005)	0.215 (0.055)	0.158 (0.056)	0.108 (0.036)
MDA	0.085 (0.028)	0.064 (0.018)	0.052 (0.011)	0.175 (0.056)	0.139 (0.047)	0.105 (0.031)
FDA	0.044 (0.014)	0.036 (0.007)	0.032 (0.004)	0.095 (0.056)	0.066 (0.032)	0.050 (0.015)
LR	0.043 (0.013)	0.034 (0.006)	0.031 (0.003)	0.098 (0.060)	0.063 (0.034)	0.045 (0.014)
GAM	0.107 (0.059)	0.053 (0.031)	0.034 (0.015)	0.186 (0.070)	0.130 (0.072)	0.071 (0.041)
RF	0.053 (0.012)	0.046 (0.010)	0.041 (0.008)	0.151 (0.045)	0.126 (0.034)	0.103 (0.022)
XGBoost	0.116 (0.034)	0.098 (0.024)	0.078 (0.017)	0.155 (0.052)	0.134 (0.038)	0.114 (0.027)
KT	0.053 (0.018)	0.043 (0.010)	0.038 (0.005)	0.122 (0.070)	0.087 (0.050)	0.062 (0.029)
LIU	0.206 (0.012)	0.203 (0.006)	0.201 (0.004)	0.226 (0.029)	0.224 (0.029)	0.217 (0.024)
Scenario E—linear logistic model						
TDA	0.031 (0.026)	0.014 (0.011)	0.007 (0.006)	0.055 (0.051)	0.029 (0.026)	0.014 (0.011)
TDA	0.069 (0.038)	0.033 (0.019)	0.015 (0.008)	0.232 (0.084)	0.120 (0.070)	0.051 (0.034)
lsTDA	0.068 (0.040)	0.033 (0.020)	0.015 (0.009)	0.147 (0.078)	0.080 (0.053)	0.036 (0.025)
lsTDA	0.090 (0.042)	0.047 (0.023)	0.022 (0.011)	0.242 (0.076)	0.138 (0.068)	0.065 (0.037)
sTDA	0.098 (0.041)	0.058 (0.026)	0.031 (0.012)	0.184 (0.070)	0.142 (0.062)	0.086 (0.039)
sTDA	0.109 (0.042)	0.067 (0.027)	0.037 (0.013)	0.226 (0.070)	0.174 (0.066)	0.106 (0.044)
LDA	0.028 (0.025)	0.013 (0.011)	0.007 (0.006)	0.051 (0.049)	0.027 (0.025)	0.013 (0.011)
QDA	0.074 (0.037)	0.039 (0.020)	0.019 (0.010)	0.225 (0.080)	0.124 (0.065)	0.059 (0.035)
MDA	0.088 (0.039)	0.050 (0.023)	0.027 (0.011)	0.110 (0.065)	0.069 (0.042)	0.040 (0.022)
FDA	0.028 (0.025)	0.013 (0.011)	0.007 (0.006)	0.051 (0.049)	0.027 (0.025)	0.013 (0.011)
LR	0.028 (0.025)	0.013 (0.011)	0.007 (0.006)	0.066 (0.061)	0.028 (0.026)	0.014 (0.012)
GAM	0.104 (0.066)	0.042 (0.035)	0.019 (0.015)	0.181 (0.080)	0.113 (0.089)	0.043 (0.044)
RF	0.071 (0.030)	0.057 (0.018)	0.046 (0.011)	0.110 (0.042)	0.089 (0.031)	0.071 (0.019)
XGBoost	0.118 (0.041)	0.100 (0.029)	0.082 (0.019)	0.124 (0.052)	0.102 (0.038)	0.086 (0.024)
KT	0.035 (0.031)	0.018 (0.014)	0.009 (0.007)	0.062 (0.054)	0.034 (0.031)	0.018 (0.015)
LIU	0.186 (0.035)	0.174 (0.021)	0.167 (0.012)	0.173 (0.043)	0.160 (0.034)	0.148 (0.020)
Scenario E—logistic model with interactions						
TDA	0.117 (0.033)	0.098 (0.014)	0.089 (0.007)	0.335 (0.064)	0.302 (0.062)	0.272 (0.048)
TDA	0.127 (0.037)	0.092 (0.019)	0.073 (0.010)	0.351 (0.061)	0.303 (0.063)	0.268 (0.051)
lsTDA	0.102 (0.041)	0.063 (0.020)	0.045 (0.010)	0.239 (0.122)	0.124 (0.091)	0.059 (0.041)
lsTDA	0.103 (0.040)	0.056 (0.021)	0.030 (0.011)	0.298 (0.110)	0.134 (0.092)	0.056 (0.042)
sTDA	0.139 (0.046)	0.094 (0.026)	0.063 (0.014)	0.325 (0.084)	0.236 (0.102)	0.130 (0.069)
sTDA	0.134 (0.044)	0.083 (0.025)	0.048 (0.014)	0.353 (0.071)	0.248 (0.096)	0.129 (0.069)
LDA	0.114 (0.031)	0.097 (0.014)	0.089 (0.007)	0.334 (0.063)	0.303 (0.061)	0.274 (0.049)
QDA	0.094 (0.037)	0.053 (0.020)	0.029 (0.011)	0.297 (0.106)	0.143 (0.097)	0.061 (0.045)
MDA	0.115 (0.038)	0.071 (0.022)	0.041 (0.013)	0.248 (0.135)	0.114 (0.091)	0.056 (0.034)
FDA	0.114 (0.031)	0.097 (0.014)	0.089 (0.007)	0.334 (0.063)	0.303 (0.061)	0.274 (0.049)
LR	0.114 (0.031)	0.097 (0.014)	0.089 (0.007)	0.313 (0.059)	0.291 (0.059)	0.266 (0.046)
GAM	0.152 (0.062)	0.084 (0.038)	0.051 (0.016)	0.235 (0.108)	0.172 (0.093)	0.109 (0.080)
RF	0.109 (0.034)	0.081 (0.018)	0.063 (0.011)	0.109 (0.050)	0.071 (0.025)	0.055 (0.013)
XGBoost	0.160 (0.044)	0.127 (0.031)	0.097 (0.020)	0.185 (0.074)	0.105 (0.054)	0.065 (0.023)
KT	0.119 (0.036)	0.101 (0.018)	0.091 (0.009)	0.310 (0.069)	0.277 (0.059)	0.249 (0.041)
LIU	0.307 (0.011)	0.305 (0.010)	0.303 (0.009)	0.121 (0.056)	0.111 (0.039)	0.102 (0.021)

## Appendix E Model Selection and Assessment

### Model Selection

To determine the required model flexibility and evaluate performance against competing methods for hepatocellular carcinoma diagnosis we employed a repeated holdout validation procedure. This involved comparing various methods suitable for combining multiple biomarkers into an optimal diagnostic test. We computed the out‐of‐sample AUC for each method for the hepatocellular carcinoma (HCC) data. Details on these methods are given in Section 4 and on the dataset in Section 5 of the main text.

The repeated holdout validation procedure is an unbiased procedure for model selection and minimizes the bias associated with holdout validation. Briefly, the steps of the procedure are as follows:

1. Randomly divide the data into two subsets of equal size: a training and holdout set.
2. For each model, estimate its parameters from the training set and using this fitted model calculate the composite score (log‐likelihood ratio or the positive class probability) on the holdout set.
3. Repeat steps 1 and 2 to obtain a distribution of out‐of‐sample AUCs (we used 1000 iterations).

The results depicted in Figure A4 reveal consistent performance across all methods, as indicated by out‐of‐sample (OOS) AUC quartiles ranging between 0.75 and 0.85. As discussed in the main text, the variance in the biomarker distributions among HCC cases was higher than among liver cirrhosis cases (control). Consequently, the subset of proposed methods (TDA, TDA ) with only a location parameter had a relatively lower performance compared to methods that incorporated scale. Similarly, since QDA can capture different marginal variances it also performed well whilst other discriminant analysis or linear combination methods lacked this capability. The random forest classification method demonstrated good performance, aligning with findings from the simulation study. The multivariate transformation model with location‐scale marginal models and a global covariance matrix (lsTDA) yielded the highest median OOS AUC, leading us to select it for the subsequent analysis.

### Model Assessment

#### Rosenblatt Transformation

For a univariate distribution Y∈ℝ, a model diagnostic technique involves plotting the empirical CDF of the probability integral transform (PIT) values and contrasting it with the CDF of the uniform distribution. Analogously, the dependent random vector $Y={\left(Y_{1},\ldots ,Y_{J}\right)}^{⊤}\in {\mathbb{R}}^{J}$ can be transformed into a uniform random vector $U={\left(U_{1},\ldots ,U_{J}\right)}^{⊤}$ by the transformation of [48]. Each of the margins $U_{j}$ are independent Uniform(0,1) random variables. This transformation is given by

$$\begin{array}{ll} & U_{1}=F_{1}\left(Y_{1}\right)\\ & U_{2}=F_{2}\left(Y_{2}|Y_{1}\right)\\ & \;\vdots \\ & U_{J}=F_{J}\left(Y_{J}|Y_{1},\ldots ,Y_{J-1}\right) \end{array}$$

where the conditional CDF are $F_{j}\left(y_{j}|y_{j-1}\right)=\mathbb{P}\left(Y_{j}\leq y_{j}|Y_{j-1}=y_{j-1}\right)$.

Our multivariate transformation model assumes that the *transformed* random vector has a multivariate normal distribution. Under this model, by the properties of the multivariate normal distribution, the conditional distributions have a univariate normal distribution, with mean and covariance structures depending on the model parameters. Thus, we can derive estimates of these conditional CDFs by

$${\hat{F}}_{j}\left(y_{j}|y_{j-1}\right)=\hat{\mathbb{P}}\left(Y_{j}\leq y_{j}|Y_{j-1}=y_{j-1}\right)=\Phi \left(\sum_{k=1}^{j}{\hat{\tilde{\Lambda }}}_{\mathrm{jk}}{\hat{h}}_{j}\left(y_{j}\right)\right)$$

where ${\hat{h}}_{j}\left(y_{j}\right)$ represents the estimated transformation functions for j=1,…,J, evaluated at the j th biomarker value and ${\hat{\tilde{\Lambda }}}_{\mathrm{jk}}$ is the element in the j th row and k th column of the estimate of $\tilde{\Lambda }$ our model is parameterized with. Consequently, we employ the Rosenblatt transformation, using the modeled conditional distribution functions to map the original biomarker measurements to uniformity.

Figures A5 and A6 show the empirical CDFs of each of the transformed conditional margins ${\hat{F}}_{j}\left(Y_{j}|Y_{1},\ldots ,Y_{j-1}\right)$ in comparison to the theoretical CDF of the uniform distribution for cases without and with HCC, respectively. Additionally, for each margin, a Kolmogorov‐Smirnov test for testing uniformity is conducted and the corresponding p‐values are reported on the figure.

#### Monotonicity Checks

Parametric copula models are restricted in capturing different types of dependencies. Unreliable estimates may arise if any of the transformation functions lacks monotonicity. To address this, we use thin plate splines in sequential univariate additive transformation models [61] to estimate the transformations $g_{\mathrm{jk}}$ (which, under a Gaussian copula model, are $g_{\mathrm{jk}}=\Lambda_{\mathrm{jk}}h_{j}\left(y_{j}\right)$, see above) in the model

$${\hat{F}}_{j}\left(y_{j}|y_{j-1}\right)=\Phi \left(\sum_{k=1}^{j}g_{\mathrm{jk}}\left(y_{j}\right)\right)\;\text{for}\;j=2,\ldots ,J.$$

Figures A7 and A8 plot the estimated transformation functions for 2≤k<j≤4 as a function of the conditioned biomarker values in subjects without and with hepatocellular carcinoma, respectively. We use these plots to check if there are any violations of monotonicity, since the sum of monotone functions is also monotone.

In most cases, this assumption appears to hold, particularly when considering the adequate estimation of spline terms with sufficient data. However, the regression of the OPN biomarker suggests that extrapolating the model to larger biomarker values may result in unreliable estimates. A comprehensive validation study is essential before implementing the model for clinical use.

## Appendix F Additional Results

To complement the main analysis, Figure A9 displays the estimated ROC curve for the optimal combination of AFP, PIVKA‐II, OPN, and DKK obtained under TDA with location‐scale marginals and a common covariance structure. Pointwise 95% confidence bands were constructed using the parametric bootstrap procedure described in the main text, based on 1,000 bootstrap replications.

Figure A10 depicts the estimated marginal transformation functions $h_{1},\ldots ,h_{4}$ from the lsTDA model of HCC biomarkers. These transformation functions can be used in combination with the coefficients from Table 1 to calculate log‐likelihood ratio score for a new subject. Figure A11 shows the estimated covariate‐dependent AUCs segmented by age and etiological groups for HCC biomarkers using a random forest. These results are presented for comparative analysis against our method, as depicted in Figure 5, serving as a sensitivity check between the two methods.
